# Satellite NO_2_ retrievals suggest China has exceeded its NO_x_ reduction goals from the twelfth Five-Year Plan

**DOI:** 10.1038/srep35912

**Published:** 2016-10-27

**Authors:** Benjamin de Foy, Zifeng Lu, David G. Streets

**Affiliations:** 1 Saint Louis University, Department of Earth and Atmospheric Sciences, St. Louis, MO 63108, USA; 2 Argonne National Laboratory, Energy Systems Division, Argonne, IL 60439, USA

## Abstract

China’s twelfth Five-Year Plan included pollution control measures with a goal of reducing national emissions of nitrogen oxides (NO_x_) by 10% by 2015 compared with 2010. Multiple linear regression analysis was used on 11-year time series of all nitrogen dioxide (NO_2_) pixels from the Ozone Monitoring Instrument (OMI) over 18 NO_2_ hotspots in China. The regression analysis accounted for variations in meteorology, pixel resolution, seasonal effects, weekday variability and year-to-year variability. The NO_2_ trends suggested that there was an increase in NO_2_ columns in most areas from 2005 to around 2011 which was followed by a strong decrease continuing through 2015. The satellite results were in good agreement with the annual official NO_x_ emission inventories which were available up until 2014. This shows the value of evaluating trends in emission inventories using satellite retrievals. It further shows that recent control strategies were effective in reducing emissions and that recent economic transformations in China may be having an effect on NO_2_ columns. Satellite information for 2015 suggests that emissions have continued to decrease since the latest inventories available and have surpassed the goals of the twelfth Five-Year Plan.

China’s rapid economic growth has been accompanied by an increase in emissions of air pollutants^1^,^2^. In particular, the increases in emissions of nitrogen oxides (NO_x_) can be clearly seen using satellite remote sensing^3^,^4^,^5^ although some regions, such as the Pearl River Delta, experienced reductions since 2005 because of local emission control^6^.

Starting with the eleventh Five-Year Plan (FYP11, 2006–2010), policies were enacted to reduce emissions of sulfur dioxide from coal-fired power plants even as coal consumption and emissions of nitrogen oxides continued to rise^2^,^7^,^8^. These reductions were clearly identified using satellite remote sensing^9^,^10^. Inventories separated by activity sectors suggest that industrial sources and power generation account for close to three quarters of emissions and have the greatest potential for emissions reductions^11^. Increasing levels of fine particulate matter (PM_2.5_) are a possible confounding factor in the satellite detection of NO_2_ due to elevated levels in numerous cities in China^12^. Analysis of Aerosol Optical Depth (AOD) from the Moderate Resolution Imaging Spectroradiometer (MODIS) found an increasing trend from 2004 to 2007 followed by a more gradual decrease from 2008 to 2013^13^. However these trends are less pronounced than the NO_2_ trends in this study and are consequently unlikely to account for the NO_2_ trends reported here.

During the twelfth Five-Year Plan (FYP12, 2011–2015), China set a goal of reducing the annual national NO_x_ emissions by 10% compared to the year 2010 (see http://news.xinhuanet.com/politics/2011-03/16/c_121193916.htm (in Chinese)). Scenario analysis suggests that NO_x_ emissions could be reduced by 32% relative to business as usual by FYP11 and FYP12^14^. Further studies suggest reductions of emissions of 40% by the year 2020 relative to 2010^15^, and a return to levels not seen since 1995 by 2030^16^.

To achieve the FYP12 goal, China has implemented a series of stringent measures and aggressive emission standards for coal-fired power plants (GB 13223-2011, July 2011), and heavy industries such as coking (GB 16171-2012, June 2012), iron and steel (GB 28662-2012, GB 28663-2012, GB 28664-2012, June 2012), cement (GB 4915-2013, December 2013), aluminum (GB 25465-2010, September 2010), lead and zinc (GB 25466-2010, September 2010), copper, nickel, and cobalt (GB 25467-2010, September 2010), etc. ref. 17 (where GB, Guo Biao, is the Chinese National Standard). Among them, NO_x_ emission-control measures on coal-fired power plants are the most effective ones. China had already shut down most coal-fired power units with capacity less than 50 MW by the end of 2003, and began to gradually close units with capacity less than 200 MW in 2007^18^. Newly constructed units are required to be at least 300 MW and most of them have installed NO_x_ emission control devices such as low NO_x_ burners (LNB, 40–60% NO_x_ removal rate) and selective catalytic/non-catalytic reduction systems (SCR/SNCR, 90% NO_x_ removal rate). From 2011 to 2014, the installation rate of SCR/SNCR systems in coal-fired power units increased dramatically from 16.9% to 83.2% in terms of capacity^19^,^20^. As a result, net NO_x_ emission factor of the coal-fired power sector decreased sharply by 60% in only three years^17^.

Moreover, China is continuously tightening its vehicle emission standards which are a major challenge for NO_x_ emission control in part because of the difference between engine testing conditions and real-world operating conditions^21^. The China IV Emission Standard which is equivalent to Euro IV standard has been implemented since 2013; the China V Emission Standard which is equivalent to Euro V standard has been implemented in Beijing, Shanghai, and cities in Pearl River Delta, and will be enforced at the country level in 2017.

The Ozone Monitoring Instrument has been widely used to estimate trends and emissions of NO_x_ all over the world in the past decade^22^. Over China, in addition to the studies cited above, NO_2_ retrievals from the Global Ozone Monitoring Experiment-2 (GOME-2) were reconciled with OMI retrievals and were used to estimate seasonal and weekday variations^23^. Recent work found rapid growth over western China continuing through 2013^17^,^24^ whereas reductions have been observed since 2011 in the main Chinese cities as well as in the North China Plain^25^,^26^. Changes in NO_2_ are leading to shifts in the ozone chemical regime and to changes in formaldehyde levels depending on the regional conditions in China^27^.

Recently, we used a multiple linear regression model to study annual changes in NO_2_ columns over polluted areas while taking into account meteorology, pixel resolution, seasonality and day of week effects^28^. By using this method, we successfully identified the effects of the Great Recession in urban areas in the United States of America. In this work, we apply the method to China to evaluate the impact of the most recent Five-Year Plan at 18 locations shown in Fig. 1 and listed in Table 1. Although there are limited surface data to evaluate the long-term trends in China, previous work over the United States of America has found that the results from OMI columns are in agreement with surface measurements^29^. Our results confirm the announcement in November 2015 by China’s Ministry of Environmental Protection (MEP) that China has achieved its national NO_x_ emission reduction goal six months ahead of the FYP12 (http://news.xinhuanet.com/fortune/2015-11/29/c_1117292909.htm (in Chinese)).

## Results and Discussion

**Figure 2:**
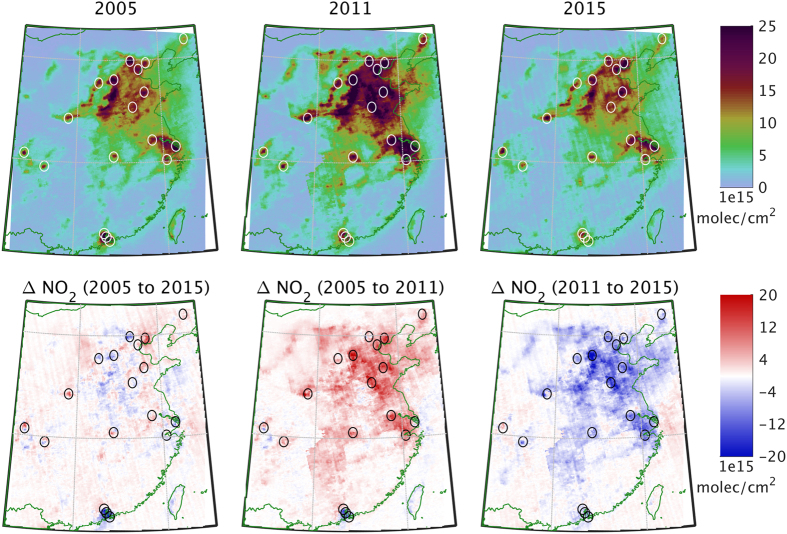
Top: Map showing the average OMI NO_2_ tropospheric Vertical Column Density for 2005 (left), 2011 (center), and 2015 (right). Bottom: Map showing the change in average OMI NO_2_ tropospheric Vertical Column Density from 2005 to 2015 (left), from 2005 to 2011 (center), and from 2011 to 2015 (right). OMI pixels were selected within the circles shown for each area. Maps generated using Matlab R2014b (http://www.mathworks.com).

Annual averages of OMI columns over China show the high NO_2_ vertical tropospheric columns over the megacities (Beijing, Yangtze Delta, Pearl River Delta) as well as industrial areas in the North China Plain (Fig. 2). Annually-averaged NO_2_ vertical columns reach values of up to 40 × 10^15^ *molec/cm*^2^. Additional hotspots can be clearly seen for Xi’an, Chengdu, Chongqing and Wuhan. The figure shows that most places experienced an increase from 2005 to 2011 followed by a decrease such that levels in 2015 are similar to 2005. In terms of net changes from 2005 to 2015, the most striking difference is the reduction in vertical columns over the Pearl River Delta, with peak annual averages decreasing from 35 × 10^15^ *molec*/*cm*^2^ to 20 × 10^15^ *molec/cm*^2^. While most of the other areas went through the increase to 2011 and decrease to 2015, the net change varies between positive and negative from site to site. There are lesser reductions visible in other cities and in the North China Plain.

We performed a multiple linear regression for each of the areas circled in Fig. 2 and listed in Table 1. The individual time series of the OMI pixels and the regression are shown for each area in the supplementary information, along with the regression parameters (Figs S6 to S23). There are between 3,000 and 7,000 data points per area, and the correlation coefficients of the fit varies between 0.61 and 0.81.

**Figure 3:**
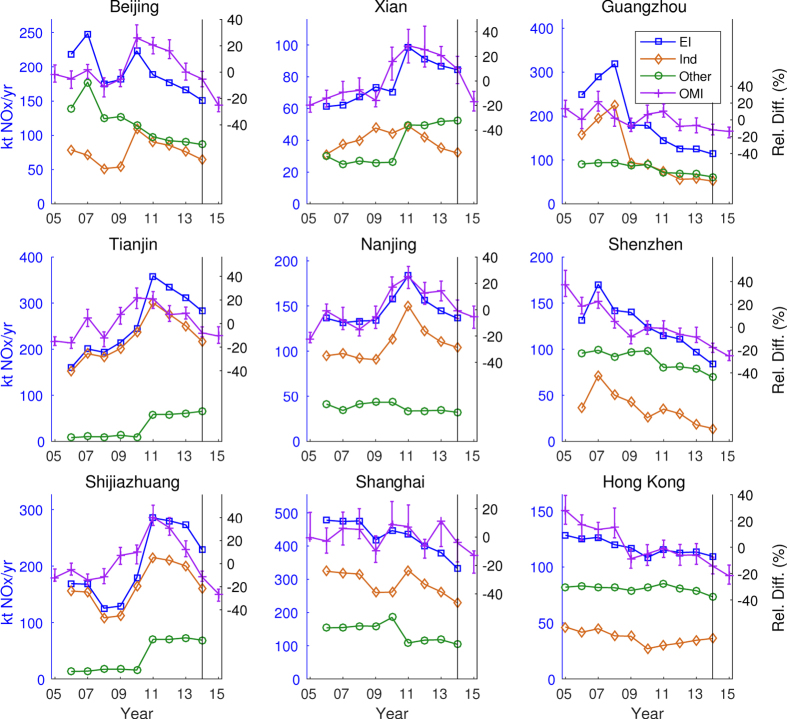
Left axes: annual trends in the emissions inventory (EI) of NO_x_ for 2006 to 2014 including industrial and power plant emissions (“Ind”) and residential and transportation emissions (“Other”). Right axes: relative percentage difference in both the emissions inventory (EI) and the OMI NO_2_ columns from the multiple linear regression model for 2005 to 2015 compared with the average over all years. Uncertainty in OMI trends shown as the full range of results from 100 bootstrapped simulations. See Fig. S1 for remaining sites in the study.

The annual variation of the official emission inventories for 2006 to 2014 are shown in Fig. 3 for the 9 sites that will be discussed below. The remaining 9 sites are shown in Fig. S1. The figures show both the total emissions and the split between industrial emissions (which includes both industry and power plants) and “Other” emissions which includes transportation and residential sources. For most areas there are gradual increases until about 2009 or 2010 and a sharp increase for 2011. This is then followed by a gradual decrease from 2012 to 2014. The right axis shows the corresponding percentage relative differences of the emissions for each year of the time period (i.e. the percentage that each year is above or below the 9-year mean).

The percentage relative difference of the emission inventories can be compared directly with the percentage relative difference of the NO_2_ vertical columns based on the scaling factors obtained from the multiple linear regression model. The graph shows the uncertainty in the results as the range from 100 realizations of the model with bootstrapping. The average range by site varies between 12 and 23% over the 11-year time period. The average standard deviation of the trends varies between 2.5 and 4.4%, which suggests that the results are robust with respect to data selection, instrumental errors and model errors. Figs S2 and S3 show the comparison of the annual trends in the satellite columns by season. The regression model was run separately for April to October (Summer) and for November to March (Winter). Because aerosol loadings vary by season^12^ as well as NO_x_ lifetimes, comparing the results by season serves as a test on the sensitivity of the method in addition to the bootstrap method. There is very good agreement between the summer, winter, and annual trends which suggests that the results are robust with respect to NO_x_ lifetimes and photochemical activity.

The correlation coefficients between the emission inventories and the vertical columns are shown in Table 1 and Fig. S4, along with the years of maximum vertical NO_2_ columns at each site. Overall, there is considerable agreement between the NO_2_ columns and the NO_x_ emissions which provides supporting evidence for the increase in emissions up until 2011 followed by a strong decrease from 2011 to 2015. In particular, Xi’an, Chongqing, Nanjing and Hangzhou have very good agreement (Pearson’s correlation coefficients between 0.86 and 0.95) with similar trends between the official inventories and the satellite retrievals.

Although data from surface measurements were not available to perform an evaluation of the trends similar to that made elsewhere^29^, Fig. S5 shows the OMI results compared with annual averages of surface measurements of NO_2_ concentrations. For the Pearl River Delta this shows that there is strong agreement for the continuous decrease in NO_2_ levels. Other sites have a discontinuity in 2013 due to an increase in data availability, but they nonetheless show a strong decrease in concentration since then that is consistent with the results from the OMI analysis.

The sites shown in Fig. 3 can be considered in 3 broad categories: the Pearl River Delta where emissions are dominated by transportation and residential (“Other”) emissions, the North China Plain where emissions are dominated by industrial emissions, and some sites where neither category dominates the other (Beijing and Xi’an).

The Pearl River Delta includes Guangzhou, Shenzhen, and Hong Kong along with other cities. Prior tests in North America were not able to identify possible differences in trends within individual urban areas^28^. While the Pearl River Delta is much larger than most urban areas in North America, the NO_2_ plume in the area is merged into a single air mass, and so there is overlap in the results from the individual cities. Nonetheless, there is a good match between the OMI results and the emissions inventory for Shenzhen with a gradual decrease over the last decade which is also seen in the measurements (see Fig. S5). In Guangzhou, there is a drop of around 60% in the emissions inventory which is not reflected in the columns. This suggests that the high emissions reported for 2006, 2007 and 2008 are not in agreement with the satellite results.

For Hong Kong, the columns overestimate the drop in the emissions inventory. Additional multiple linear regression tests were performed for the Pearl River Delta using ozone time series from Hong Kong. There was no clear trend in the ozone levels, and the inclusion of these measurements did not change the results of the regression analysis substantially. This suggests that changing levels of photochemical activity in the area are not responsible for the trends observed in the satellite columns. The drop in Hong Kong is therefore most likely related to the regional drop in emissions in the whole of the Pearl River Delta. The reasons for the significant drop in emissions in the Pearl River Delta are not entirely clear but are most likely the result of a longer history of pollution control on numerous different sources resulting from the region’s status as the most economically developed region in the country^6^.

The industrial sites in the North China Plain share a common trend with increases in emissions until 2011 and decreases since then. This is clearly visible in both Tianjin and Shijiazhuang, although the drop in emissions in 2008 and 2009 that is clearly visible in the official inventory for Shijiazhuang is not as evident in the satellite results. Tangshan (Fig. S1) is similar to Tianjin except that the columns increased before the emission inventory which suggests that there may have been a delay either in updating the inventory or in the implementation of control equipment.

Three more sites can be considered in connection with the industrial sites: Xi’an, Nanjing and Wuhan. Xi’an has similar emissions from the industrial and “Other” sectors, but the trends in the industrial emissions follows the same pattern as the other industrial sites, and appear to be the driver for the recent decrease in NO_2_ columns. Although located further south, Nanjing is also dominated by industrial emissions and follows the same industrial pattern with strong reductions since 2011.

**Figure 4:**
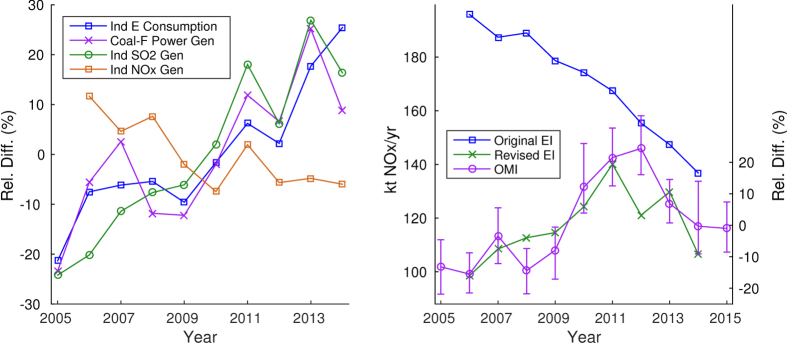
Left: Annual trends in industrial energy consumption, coal-fired power generation, industrial SO_2_ generation and industrial NO_x_ generation for Wuhan from 2005 to 2014, shown as relative percentage differences. Right: Comparison of original and revised annual emissions inventory with the trends from the OMI columns. Emission totals shown on the left axis, relative percentage difference of the revised EI and the OMI trends shown on the right axis (see also Fig. 3). Uncertainty in OMI trends shown as the full range of results from 100 bootstrapped simulations.

The largest discrepancy between the emission inventory and the OMI columns is for Wuhan (Fig. S1), where the official emission inventory shows a fairly constant decrease of over 35%, whereas the satellite results show a large increase until 2012 followed by a sharp drop. To explore the possible reasons for the discrepancies, we reviewed the relevant official statistics reported by the Wuhan Bureau of Statistics^30^. Figure 4 shows relative percentage differences of industrial activity for Wuhan including industrial energy consumption, coal-fired power generation, industrial SO_2_ generation^31^, and industrial NO_x_ generation^31^. In general, industrial SO_2_ and NO_x_ generations should be in line with the industrial activities. This is true for SO_2_ generation which increased by 54% during 2005–2014 and is in good agreement with the trends of industrial energy consumption (r = 0.89) and coal-fired power generation (r = 0.88). However, the official NO_x_ generation shows an unreasonable decrease trend over the same time period. We therefore speculate that there might be some issues in compiling the NO_x_ generation, and consequently the NO_x_ emissions, for Wuhan in the official statistics. A new estimate of NO_x_ emissions was made by using the SO_2_ generation trend combined with the official NO_x_ removal rate^31^. As can be seen in the right panel of Fig. 4, the corrected NO_x_ emission trend for Wuhan has a much better agreement with the results from the OMI columns. The above analysis implies that the top-down satellite NO_2_ observations may help us identify the potential problems in official bottom-up NO_x_ emission estimates.

Most of these industrial sites have a discontinuity in the “Other” emissions due to a change in 2011 of the estimation of emissions from transportation^31^, but this is a minor contributor relative to industrial emissions and does not offset the downward trends in NO_2_ columns. Overall, this provides evidence that the emission control standards listed in the introduction are having a significant impact on NO_2_ levels.

While Shanghai is dominated by industrial emissions, the OMI trend is weak and contains short-lived decreases for 2009 and 2012. Shanghai however is in a more complex geographic region, because of the Yangtze River Delta and the heterogeneous location of surrounding sources. Whereas the NO_2_ plume in the Pearl River Delta is fairly uniform, the heterogeneity in the Yangtze River Delta suggests that the trends may be less reliable in this case.

Beijing has similar emissions from the industrial and “Other” sectors. The industrial emissions decreased in connection with the 2008 Olympics, and increased after that before decreasing since their peak in 2010. There is a relatively poor fit between the OMI results and the emission inventory. This seems to be because of the spike in “Other” emissions in 2007. The official emissions seem to overestimate the decrease from 2006 to 2009, but correctly identify the jump in emissions in 2010. Both the columns and the retrievals have had reductions of around 30% between 2010 and 2014.

**Figure 5:**
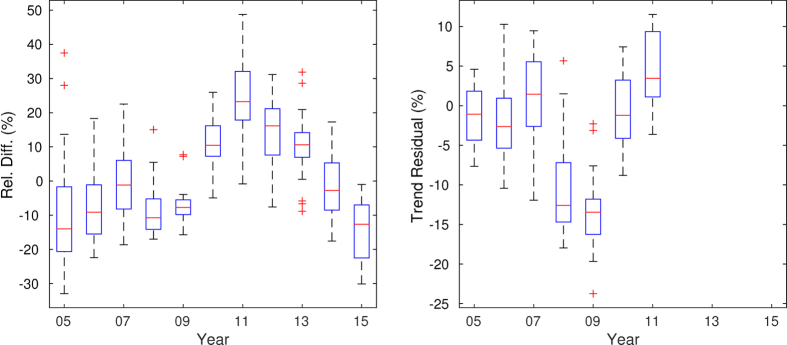
Left: Boxplot of annual percentage relative difference of OMI annual factors for all sites in this study. Right: Boxplot of the residual for each year between 2005 and 2011 relative to a linear trend for that time period.

Many sites experienced a reduction in NO_2_ columns during 2008 and 2009, which was the time of the Great Recession as well as of the 2008 Summer Olympic games in Beijing. Figure 5 shows a boxplot of the relative percentage difference of all the sites in this study, showing a clear reduction during 2008 and 2009 compared with a strong rising trend from 2005 to 2011. The right panel shows a boxplot of the residual of the percentage relative difference compared with a linear trend for each station from 2005 to 2011. In this way, the drop during 2008 and 2009 can be estimated separately from the different trends at each site. This shows that the interquartile range of reductions was between 7 and 15% in 2008 and between 12 and 16% in 2009. Although most sites had lower columns in 2009 than 2008, the reverse is true for Beijing. This may be associated with the air quality control measures implemented for the 2008 Summer Olympic games. Because the recession and the Olympic games overlap, it is not possible to clearly distinguish between their impacts. Nevertheless, the decrease in NO_2_ columns is not limited to the areas of the country that had special air quality policy measures for the Olympic games.

Table 1 shows the percentage relative difference in the NO_2_ columns between the maximum year and 2014. These show reductions of around 30% in most of the main urban areas and reductions between 0 and 20% in the more industrial regions. Also shown are the relative differences from 2014 to 2015. These are much stronger in the industrial areas, with decreases of up to 24%, and continue to be sizable in the urban areas with columns decreasing in many areas by around 10% or more. These estimates are in agreement with the first annual report on the environment to the Standing Committee of the National People’s Congress (http://news.xinhuanet.com/english/2016-04/25/c_135311023.htm) which report NO_x_ average national reductions of 19% from 2010 to 2015 and 11% from 2014 to 2015.

A contributing factor to the recent decrease in NO_2_ columns is the economic slowdown that has taken place since 2015. Gross Domestic Product growth was 6.9% in 2015, which is the lowest growth rate since 1990. This is combined with a shift from an investment-driven economy towards a more consumer-driven economy. Additionally, the Chinese government has been putting great efforts in reducing industrial overcapacity since 2015, particularly in the coal and steel industries which typically have high NO_x_ emissions. While we cannot distinguish the relative impacts of pollution control policies and economic transformations at this time, the results clearly suggest that emissions of NO_x_ are continuing to decrease considerably over most of the areas in China that have large vertical NO_2_ columns.

## Summary

We analyzed retrievals of the vertical column density of NO_2_ from the Ozone Monitoring Instrument (OMI) over an 11 year period from 2005 to 2015 in China. The estimated trends from the satellite remote sensing were compared with official annual emission inventories. There was an excellent agreement in most cases which increases the confidence in the quality of the trends derived from both the official NO_x_ emission estimates and the satellite products. Comparisons with the official emission inventories of industrial and power plant sources suggest that these have been the main drivers of the recent reductions. Reductions are most likely the result of the combination of pollution control policies and recent economic transformations. Overall, China is on track to achieve NO_x_ emissions consistent with previously described successful policy scenarios^16^.

In some cases, discrepancies can be used to identify specific areas needing further study. By comparing the annual columns in 2008 and 2009 with the linear trends for the areas considered, it was possible to estimate that the combination of clean air policies for the 2008 Beijing Summer Olympic games and the Great Recession was accompanied by reductions of up to 20% in NO_2_ columns. We further expect that improved numerical simulations of atmospheric chemistry can be obtained for current time periods by applying scale factors obtained from satellite remote sensing. Overall, the results showed that China had already met its NO_x_ reduction goals set out in the twelfth Five-Year Plan by 2014, and that reductions continued in 2015.

## Methods

### OMI Retrievals

The Ozone Monitoring Instrument (OMI) is a Dutch-Finnish instrument launched on NASA’s Aura satellite in July 2004 and has been providing measurements of ultraviolet and visible radiation down to a resolution of 13 km by 24 km^32^. This paper uses the Vertical Column Density (VCD) of tropospheric NO_2_ from the DOMINO v2.0 product^33^,^34^.

We selected 18 areas in China as shown in Table 1 and Fig. 2. The areas cover most of the NO_2_ hotspots in China. We also performed the analysis for additional industrial hotspots in the southwest of the North China Plain, but do not report them here as the results are similar to the neighboring sites reported in this paper. For each area selected, we retrieved all level 2 swath pixels at the native instrument resolution within a 0.5 degree radius of the center point.

OMI has suffered from a partial blockage of its field of view leading to row anomalies. In order to have a consistent record, we limit the analysis to rows 11 to 23 for the years from 2005 to 2015 inclusive. Only data points with a quality assurance flag of 0 were retained which excludes all data points with a cloud radiance fraction greater than 50%. Furthermore, all points with a surface albedo exceeding 30% were excluded, as were all points with a solar zenith angle greater than 70 degrees. Overall, we have between 3,000 and 7,000 OMI pixels for each of the 18 areas selected over the 11-year period being analyzed.

### Multiple Linear Regression

We use a multiple linear regression method specifically developed to analyze OMI pixel data^28^ which is similar to other models developed for OMI^35^,^36^,^37^. Because we are working with the original OMI pixel data with no averaging, we have a long time series for each site and can use the regression model to account for weekly, seasonal, and annual variations as well as the impact of meteorology and pixel resolution. Briefly, the model includes the factors listed in Table 2 and can be described by Eq. 1:


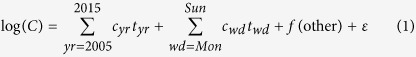


Where *C* is the series of NO_2_ columns; the coefficients *c* are determined by the Iteratively Reweighted Least Squares procedure; the time vectors (*t*_*yr*_, and *t*_*wd*_) represent the annual and weekly variation; and *ε* is the residual between the model and the retrievals. We use the natural logarithm of the OMI NO_2_ columns because they are log-normally distributed over polluted areas. *f*(other) is made up of the following components:


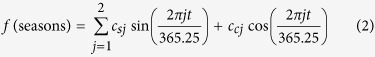



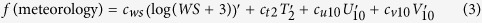



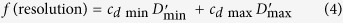


The inputs to the meteorology and resolution function are normalized variables (*T*′ = (*T* − *μ*(*T*))/*σ*(*T*)): WS is the 10 m wind speed; T_2_ is the 2 m temperature; U_10_ and V_10_ are the zonal and meridional 10 m wind speeds. The meteorological time series are obtained from ERA-Interim data at 1 degree and 6 hour resolution^38^ which are available from the European Center for Medium-Range Weather Forecasts (http://apps.ecmwf.int/datasets/). D_min_ is the distance of the nearest pixel corner to the center point of the urban area, and is negative if the urban area is inside the pixel. D_max_ is the distance of the farthest pixel corner from the center of the urban area.

In contrast with the analysis over cities in the United States^28^, we do not include a linear factor for long-term trends, but instead use a separate factor for each year in the study. We further do not consider holidays separately as their signal was not as clear as in the United States. A least squares solver is used for the multiple linear regression^39^. To account for outliers, we use an Iteratively Reweighted Least Squares (IRLS) procedure.

The uncertainty in the regression results is estimated using bootstrapping. 100 realizations of the model were performed using randomly selected days to be included in the analysis with replacement. This accounts for both measurement errors and model errors as the instrument errors are assumed to be uncorrelated and model errors are unlikely to be correlated beyond a couple of days^28^.

### Official NO_x_ Emission Inventory

We compared the trends in OMI NO_2_ columns for 18 areas with official bottom-up NO_x_ emissions. For the 17 areas in mainland China, we use the official NO_x_ emissions at the municipality level for Beijing, Tianjin, Shanghai, and Chongqing and at the prefecture city level for the other areas^31^. Mainland China began to report official NO_x_ emissions in 2006, and the data are available annually with 2014 being the most recent year currently available. The interannual trends of the official NO_x_ emissions are reported to be fairly reasonable^9^,^17^,^40^, despite the omission of emissions from rural industries and biofuels^9^,^17^,^40^,^41^,^42^. For Hong Kong, NO_x_ emissions for the period of 2005–2014 were obtained from the Hong Kong Air Pollutant Emission Inventory, which is compiled by the Hong Kong Environmental Protection Department^43^.

## Additional Information

**How to cite this article**: de Foy, B. *et al*. Satellite NO_2_ retrievals suggest China has exceeded its NO_x_ reduction goals from the twelfth Five-Year Plan. *Sci. Rep.* **6**, 35912; doi: 10.1038/srep35912 (2016).

**Publisher’s note:** Springer Nature remains neutral with regard to jurisdictional claims in published maps and institutional affiliations.

## Supplementary Material

Supplementary Information

## Figures and Tables

**Figure 1:**
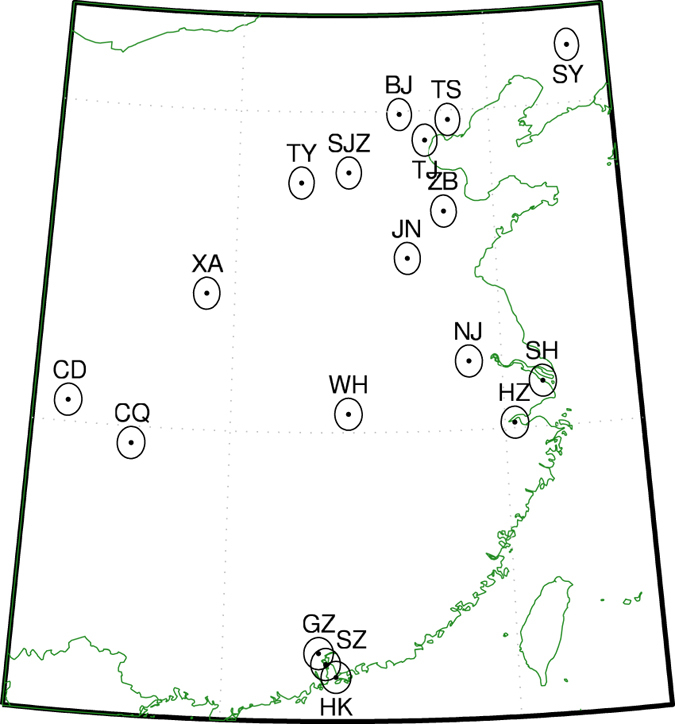
Map showing the location of the analysis sites, see Table 1 for full names. OMI pixels were selected within the circles shown for each area. Map generated using Matlab R2014b (http://www.mathworks.com).

**Table 1:** Areas included in this study along with selected parameters from the multiple linear regression analysis: mean vertical OMI NO_2_ column (VCD), number of pixel retrievals in each time series, correlation coefficient between OMI time series and MLR fit.

Location	Abbr.	Latitude	Longitude	Mean NO_2_ VCD	Pixels	r MLR	r EI/OMI	OMI Max	Δ(Max - 2014)	Δ(2014–2015)
		°N	°E	1 × 10^15^ *molec*/*cm*^2^	No.			Year	Rel. Diff. %	Rel. Diff. %
Beijing	BJ	39.90	116.40	22.83	6,797	0.80	0.27	2010	−24.7	−21.1
Tianjin	TJ	39.08	117.40	22.01	7,143	0.71	0.61	2010	−24.1	−3.1
Tangshan	TS	39.70	118.35	21.34	6,913	0.73	0.77	2011	−7.8	−16.3
Shenyang	SY	41.80	123.42	11.30	4,736	0.73	0.68	2011	−28.2	−8.2
Shijiazhuang	SJZ	38.10	114.35	25.78	6,367	0.76	0.71	2011	−37.1	−16.4
Taiyuan	TY	37.78	112.48	13.85	6,524	0.67	0.53	2011	−26.3	−24.3
Zibo	ZB	36.85	118.05	22.50	6,450	0.74	0.81	2011	−20.3	−11.5
Jining	JN	35.41	116.59	18.90	5,129	0.80	0.76	2011	−38.5	−15.3
Xi’an	XA	34.26	108.93	14.89	4,692	0.77	0.86	2011	−14.8	−24.4
Chongqing	CQ	29.48	106.45	9.97	2,942	0.79	0.95	2011	−12.8	−23.6
Chengdu	CD	30.67	104.05	12.18	2,996	0.81	0.71	2011	−1.6	−19.8
Wuhan	WH	30.58	114.30	11.93	4,907	0.81	−0.56	2012	−19.9	−0.7
Nanjing	NJ	32.15	118.78	17.42	5,025	0.73	0.89	2011	−20.7	−4.8
Shanghai	SH	31.42	121.47	21.91	5,881	0.73	0.25	2013	−13.9	−9.1
Hangzhou	HZ	30.17	120.35	17.20	5,307	0.80	0.86	2011	−15.9	−14.1
Guangzhou	GZ	23.12	113.26	15.84	4,532	0.61	0.58	2007	−27.2	−2.3
Shenzhen	SZ	22.78	113.50	18.47	4,692	0.78	0.81	2005	−40.0	−9.6
Hong Kong	HK	22.38	113.85	15.01	4,848	0.75	0.90	2005	−33.1	−8.8

In addition the table shows the correlation coefficient between the OMI trends and the emission inventory (EI) trends, the year of maximum VCD, the relative difference in OMI NO_2_ between 2014 and the maximum year and the relative difference between 2014 and 2015.

**Table 2:** Factors included in the multiple linear regression analysis of the NO_2_ columns.

Annual	Seasonal	Weekday	Meteorology	Pixel Resolution
2005–2015	Sin 1yr	Mon - Sun	Wind Speed	Min Pixel Distance
	Cos 1 yr		Zonal Wind	Max Pixel Distance
	Sin 6 mo		Meridional Wind	
	Cos 6 mo		Temperature	
