# Supplementary material for: Satellite NO2 retrievals suggest China has exceeded its NOx reduction goals from the twelfth Five-Year Plan
Source: Sci Rep. 2016 Oct 27;6:35912. doi: 10.1038/srep35912 (PMC5082360; doi:10.1038/srep35912)
Supplement: Supplementary Information [file srep35912-s1.pdf]

Satellite NO_2 retrievals suggest China has exceeded its NO_X reduction goals from the twelfth Five-Year Plan

Benjamin de Foy, Zifeng Lu and David G. Streets, Scientific Reports 2016, Supplementary Material.

Supplementary Material

This supplementary material contains extra tables and figures for: B. de Foy, Z. Lu, and D. G. Streets, "Satellite NO_2 retrievals suggest China has exceeded its NO_X reduction goals from the twelfth Five-Year Plan", Scientific Reports, 2016.

Please see the paper for data description, discussion and acknowledgments.

Annual Time Series

Table S1 shows the percentage relative difference in vertical OMI NO_2 columns for each year and for each site of the study. Figure S1 shows the annual trends in the emissions inventory and the OMI columns for the sites not shown in Fig. 3.

Figures S2 and S3 show the sensitivity of the annual trends in OMI NO_2 columns to the months of the year included in the analysis.

Maps

Fig. S4 shows a map of the correlation coefficient between the OMI annual trends and the official emissions inventory, as well as a map of the year of maximum OMI column scaling factor. Please see Table 1 for data.

Surface Measurements

Annual average NO_2 concentrations were collected from a wide range of official sources including environmental bulletins and statements of various cities and provinces; the China Environment Yearbook (China Environmental Newspaper, Beijing, China, 2006-2015); and the China Statistical Yearbook on Environment (National Bureau of Statistics, Ministry of Environmental Protection, China Statistics Press, Beijing, China, 2006-2014). The NO_2 concentration data of Hong Kong are from the air quality reports of the Hong Kong Environmental Protection Department (Air Quality Reports in Hong Kong 2005-2015. Tech. Rep. Hong Kong Environmental Protection Department, Hong Kong).

Fig. S5 shows the annual average NO_2 concentrations along with the annual trends in the OMI columns shown in Fig. 3. The implementation of the new Environmental Protection Law and the new Atmospheric Environmental Quality Standards led to hundreds of new monitoring sites built during 2012 to 2014. This explains the discontinuity in the concentrations leading up to 2013. Nonetheless the data for 2013, 2014 and 2015 should be comparable.

Multiple Linear Regression Model for Each Urban Area

Figures S6 to S23 show the multiple linear regression model for each of the 18 areas in this study along with model parameters.

Table S1: Percentage relative difference in vertical OMI NO_2 columns for each year and for each site of the study. The differences are calculated as percentages relative to the 11-year mean of the columns.

Location	2005 %	2006 %	2007 %	2008 %	2009 %	2010 %	2011 %	2012 %	2013 %	2014 %	2015 %
Beijing	-1.7	-4.9	1.5	-11.1	-5.5	26.0	20.4	15.9	0.5	-5.2	-25.2
Tianjin	-14.8	-16.5	5.2	-12.3	7.7	21.5	21.1	7.6	8.6	-7.8	-10.7
Tangshan	-33.0	-22.4	-4.2	-10.5	-7.6	21.8	25.1	21.2	18.2	15.3	-3.5
Shenyang	-19.0	-10.2	1.9	-14.7	-7.8	7.2	37.3	18.7	9.9	-1.4	-9.5
Shijiazhuang	-11.9	-5.3	-14.1	-11.7	7.2	9.7	40.3	31.2	11.9	-11.7	-26.2
Taiyuan	-4.4	5.9	7.7	-5.2	-10.2	9.9	25.3	12.2	8.0	-7.7	-30.1
Zibo	-26.6	-17.2	-10.5	-17.0	-7.4	11.0	32.1	25.8	31.9	5.3	-6.8
Jining	-20.6	-11.9	-1.9	-8.0	-13.5	13.4	48.8	19.1	28.6	-8.5	-22.5
Xi'an	-19.6	-14.2	-9.2	-7.6	-15.7	15.4	28.7	25.5	20.9	9.7	-17.0
Chongqing	-15.7	-10.3	-18.6	-14.2	-4.9	6.7	34.5	16.4	13.3	17.3	-10.3
Chengdu	-21.4	-15.6	-0.4	-12.3	-3.9	8.7	17.9	16.6	11.5	16.0	-7.0
Wuhan	-13.2	-15.5	-3.4	-14.4	-7.9	12.1	21.5	24.4	6.9	-0.3	-1.0
Nanjing	-22.3	-1.1	-8.2	-15.0	-5.5	17.3	25.0	12.8	14.2	-0.9	-5.6
Shanghai	-0.1	-2.8	6.1	5.5	-9.8	8.9	7.5	-6.4	11.3	-4.1	-12.8
Hangzhou	-9.2	-8.0	-2.9	-5.8	-12.1	16.2	21.1	10.8	7.2	1.8	-12.5
Guangzhou	13.7	0.2	21.1	2.1	-7.7	6.8	10.0	-7.6	-6.7	-11.8	-13.8
Shenzhen	37.5	18.3	22.5	5.5	-8.4	-0.4	-0.8	-6.2	-8.9	-17.6	-25.4
Hong Kong	28.0	17.3	13.5	15.0	-8.7	-5.0	-0.8	-6.1	-5.8	-14.3	-21.9

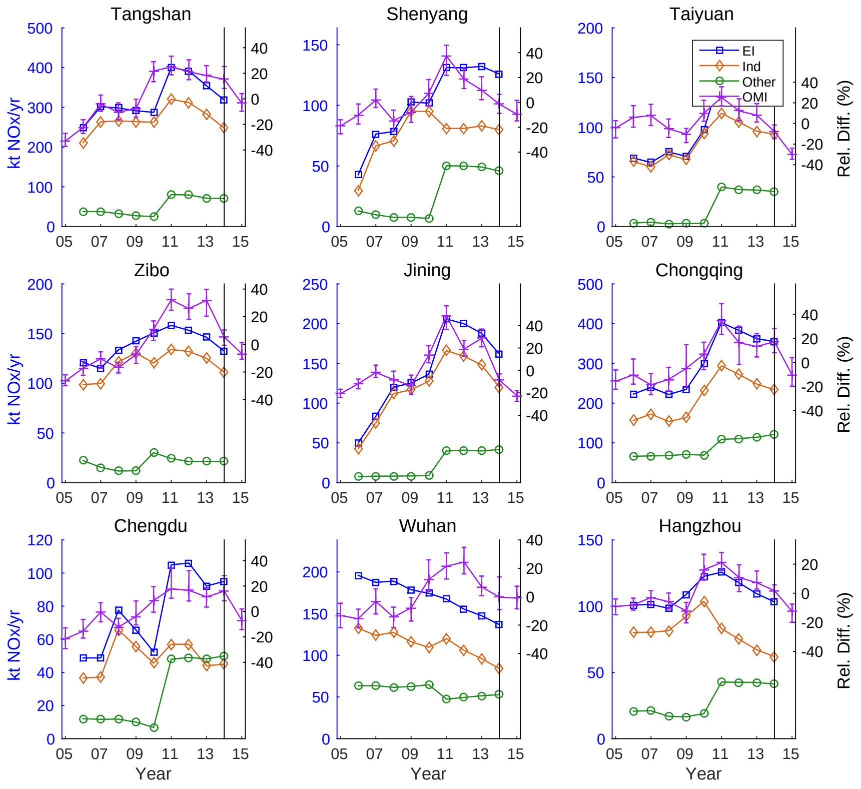

Figure S1: Left axes: annual trends in the emissions inventory (EI) of NO_x for 2006 to 2014 including industrial and power plant emissions ("Ind") and residential and transportation emissions ("Other"). Right axes: relative percentage difference in both the emissions inventory (EI) and the OMI NO_2 columns from the multiple linear regression model for 2005 to 2015 compared with the average over all years. Uncertainty in OMI trends shown as the full range of results from 100 bootstrapped simulations. See Fig. 2 for other sites in the study.

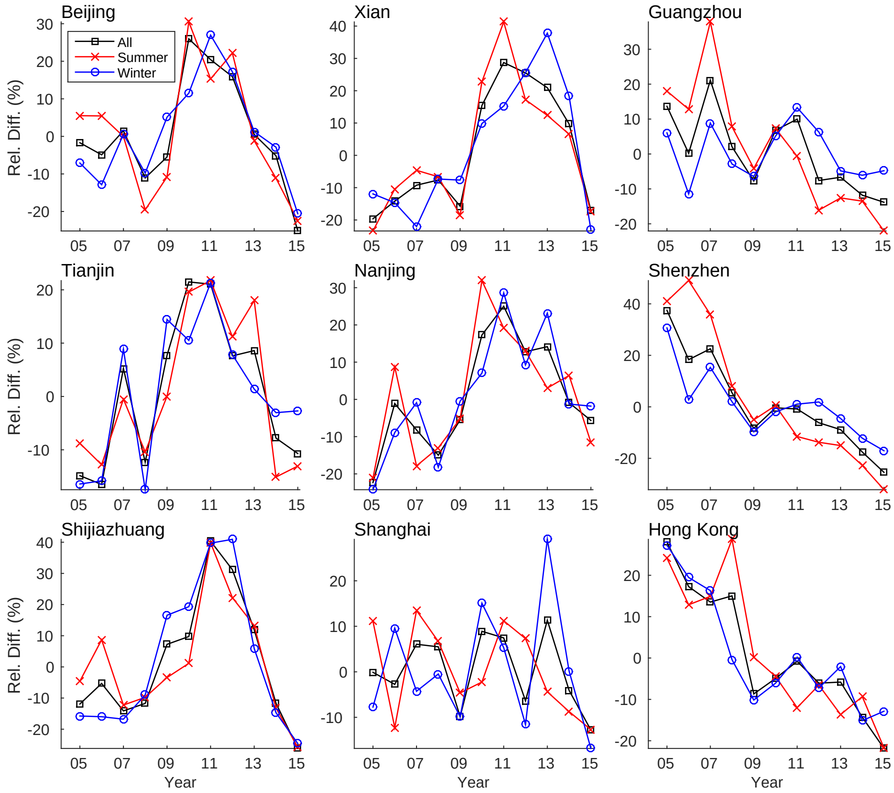

Figure S2: Annual Trends in OMI NO_2 columns for the entire time series (All), for April to October (Summer) and for November to March (Winter) for the sites shown in Fig. 2.

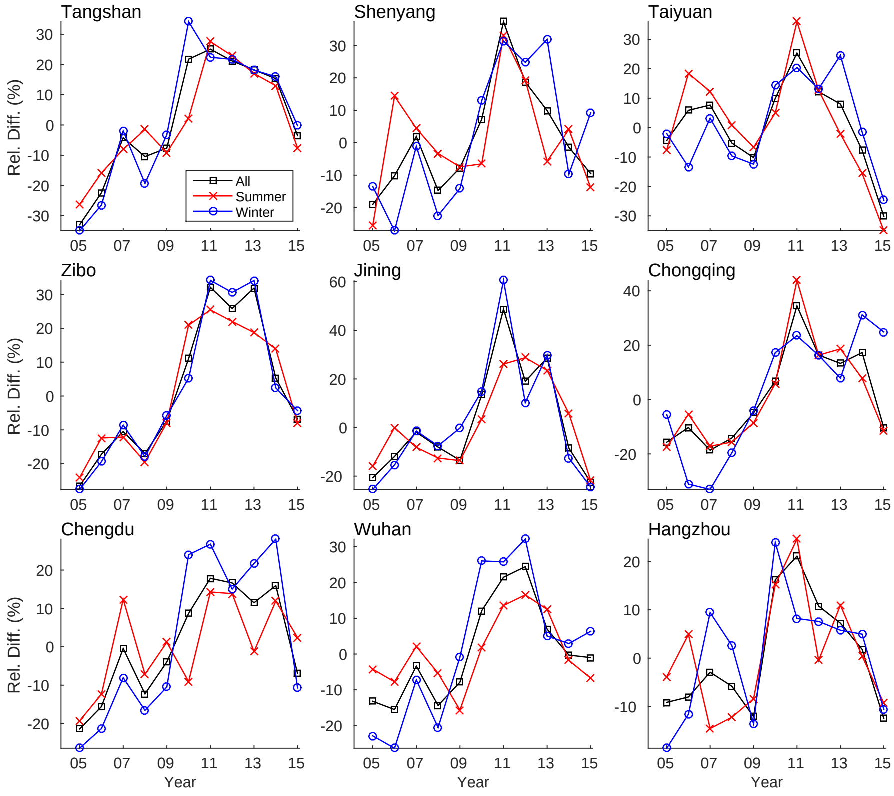

Figure S3: Annual Trends in OMI NO_2 columns for the entire time series (All), for April to October (Summer) and for November to March (Winter) for the sites shown in Fig. S1.

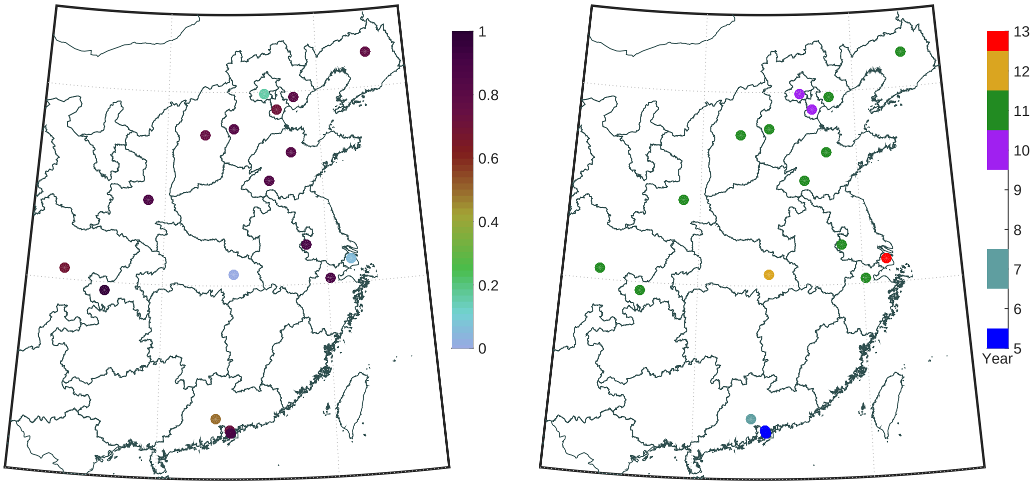

Figure S4: Left: Correlation coefficient between OMI annual trends and official emission inventories for each area. Right: Year of maximum OMI vertical NO_2 columns based on the multiple linear regression factor. (Figure generated with Matlab R2014b, http://www.mathworks.com).

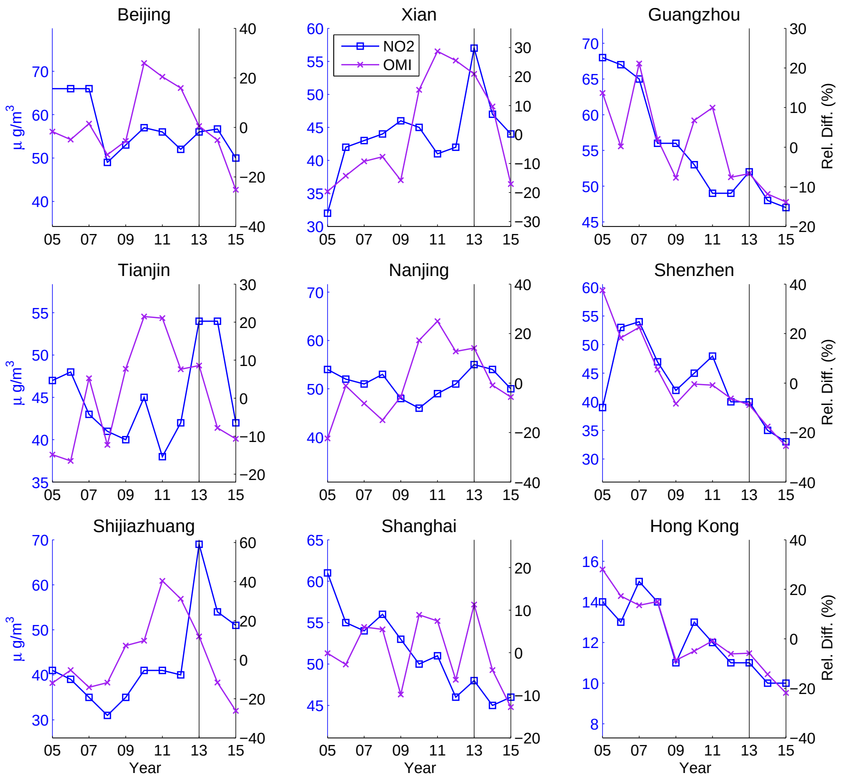

Figure S5: Left axis: Annual average NO_2 concentrations from surface measurements. Right axis: Relative percentage difference in the OMI NO_2 columns from the multiple linear regression model for 2005 to 2015 compared with the average over all years. (Figure generated with Matlab R2014b, http://www.mathworks.com).

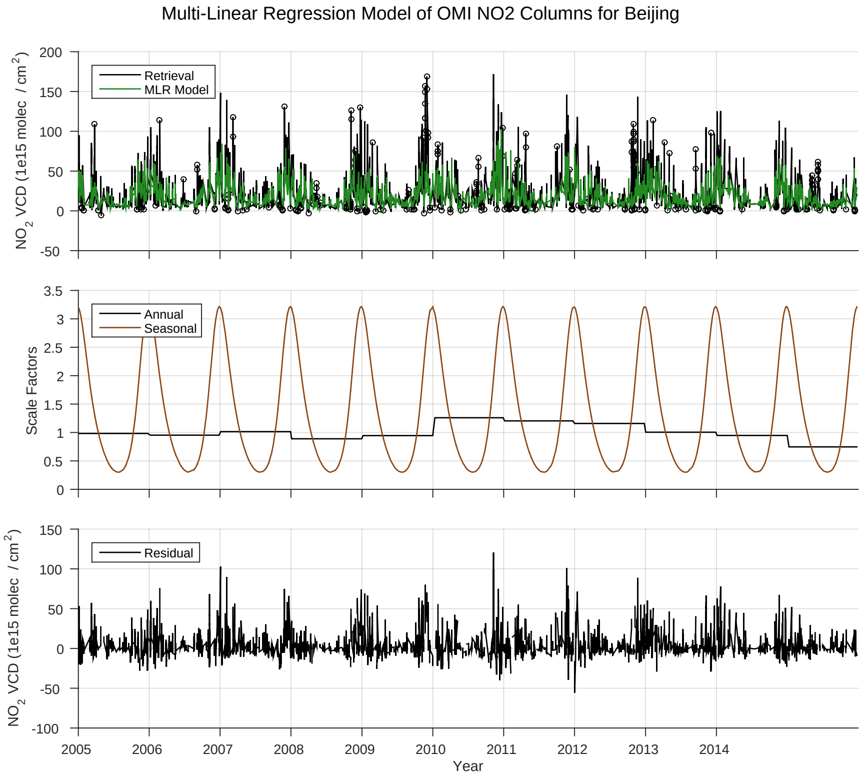

Stats		Annual	%	Weekday	%	Met/Pixel		Seasonal	
Nobs	6796	2005	-1.7	Mon	-6.9	WS10	-0.24	Sin 1yr	0.19
Noutliers	318	2006	-4.7	Tue	-11.2	T2	0.35	Cos 1yr	1.13
r	0.80	2007	1.5	Wed	0.0	U10	-0.02	Sin 6mo	-0.15
Mean VCD	22.8	2008	-11.1	Thu	8.2	V10	0.38	Cos 6mo	0.03
		2009	-5.5	Fri	4.0	Dmin	-0.07		
		2010	26.0	Sat	-7.5	Dmax	-0.13		
		2011	20.4	Sun	-7.5				
		2012	15.9						
		2013	0.5						
		2014	-5.2						
		2015	-25.3						

Figure S6: Multiple linear regression analysis for Beijing showing the time series of NO_2 OMI retrievals and the multiple linear regression model fit (top), the annual and seasonal scale factors (middle) and the residual (bottom). Outliers are shown by open circles. The table below the graphs shows the statistics for the model; the percentage difference in NO_2 Vertical Column Density (VCD) for the annual and weekday factors; and multiple linear regression factors for meteorology, pixel resolution and seasonal factors.

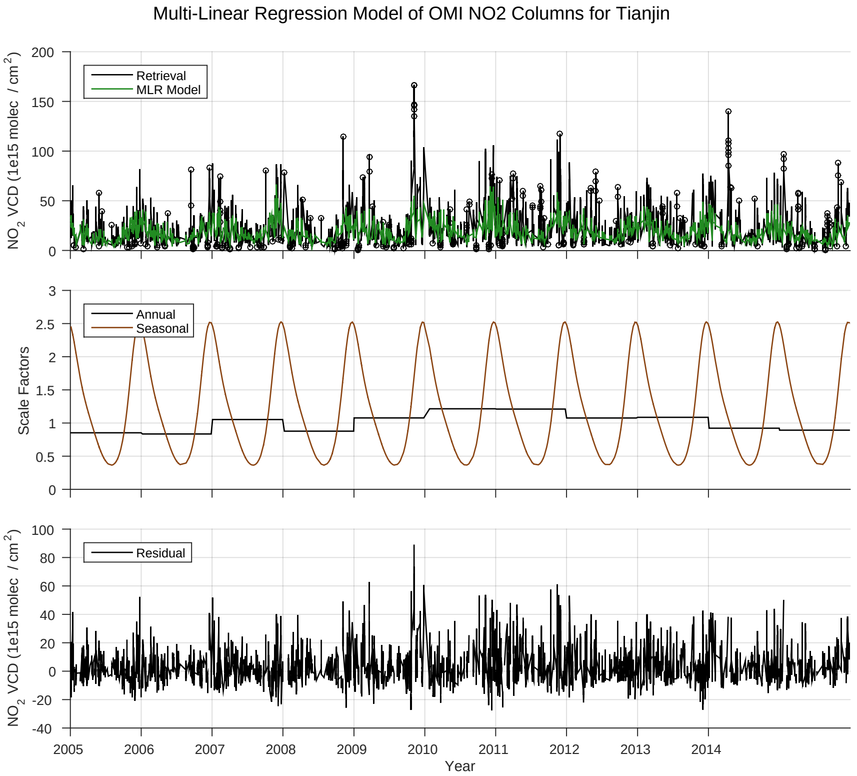

Stats		Annual	%	Weekday	%	Met/Pixel		Seasonal	
Nobs	7142	2005	-14.7	Mon	6.9	WS10	-0.15	Sin 1yr	0.18
Noutliers	326	2006	-16.5	Tue	-2.1	T2	0.28	Cos 1yr	0.89
r	0.71	2007	5.2	Wed	0.0	U10	-0.07	Sin 6mo	-0.19
Mean VCD	22.0	2008	-12.3	Thu	-1.5	V10	0.24	Cos 6mo	0.02
		2009	7.7	Fri	3.7	Dmin	-0.05		
		2010	21.5	Sat	-1.9	Dmax	-0.07		
		2011	21.1	Sun	-6.1				
		2012	7.6						
		2013	8.6						
		2014	-7.8						
		2015	-10.8						

Figure S7: Multiple linear regression analysis for Tianjin showing the time series of NO_2 OMI retrievals and the multiple linear regression model fit (top), the annual and seasonal scale factors (middle) and the residual (bottom). Outliers are shown by open circles. The table below the graphs shows the statistics for the model; the percentage difference in NO_2 Vertical Column Density (VCD) for the annual and weekday factors; and multiple linear regression factors for meteorology, pixel resolution and seasonal factors.

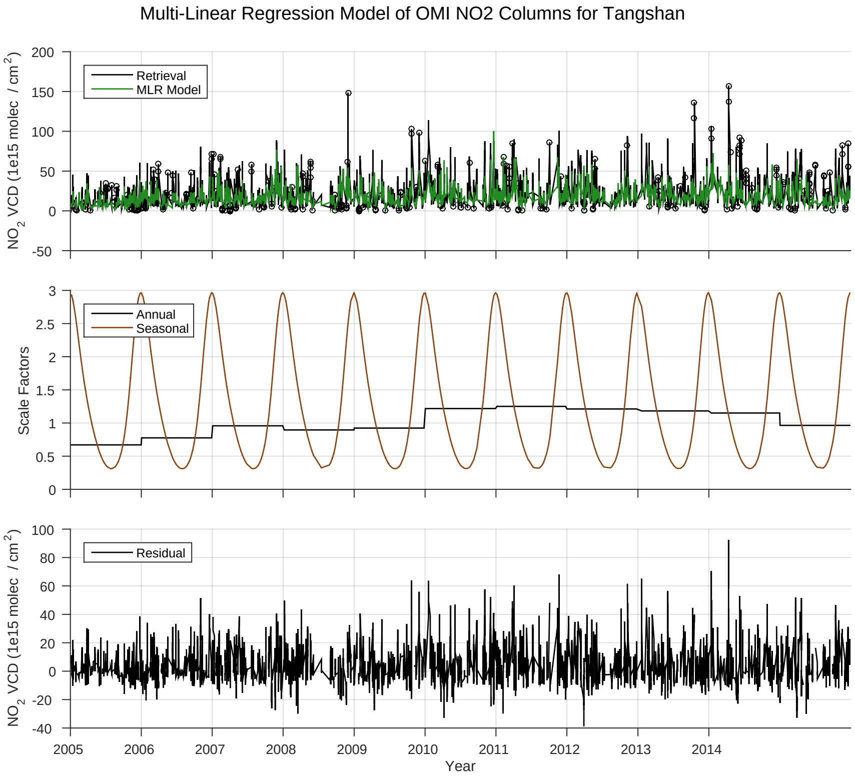

Stats		Annual	%	Weekday	%	Met/Pixel		Seasonal	
Nobs	6912	2005	-33.0	Mon	19.7	WS10	-0.19	Sin 1yr	0.27
Noutliers	302	2006	-22.4	Tue	5.0	T2	0.50	Cos 1yr	1.05
r	0.73	2007	-4.2	Wed	0.0	U10	0.04	Sin 6mo	-0.16
Mean VCD	21.3	2008	-10.4	Thu	-0.9	V10	0.33	Cos 6mo	0.03
		2009	-7.6	Fri	-3.9	Dmin	-0.13		
		2010	21.8	Sat	7.3	Dmax	-0.08		
		2011	25.1	Sun	3.5				
		2012	21.2						
		2013	18.3						
		2014	15.1						
		2015	-3.5						

Figure S8: Multiple linear regression analysis for Tangshan showing the time series of NO_2 OMI retrievals and the multiple linear regression model fit (top), the annual and seasonal scale factors (middle) and the residual (bottom). Outliers are shown by open circles. The table below the graphs shows the statistics for the model; the percentage difference in NO_2 Vertical Column Density (VCD) for the annual and weekday factors; and multiple linear regression factors for meteorology, pixel resolution and seasonal factors.

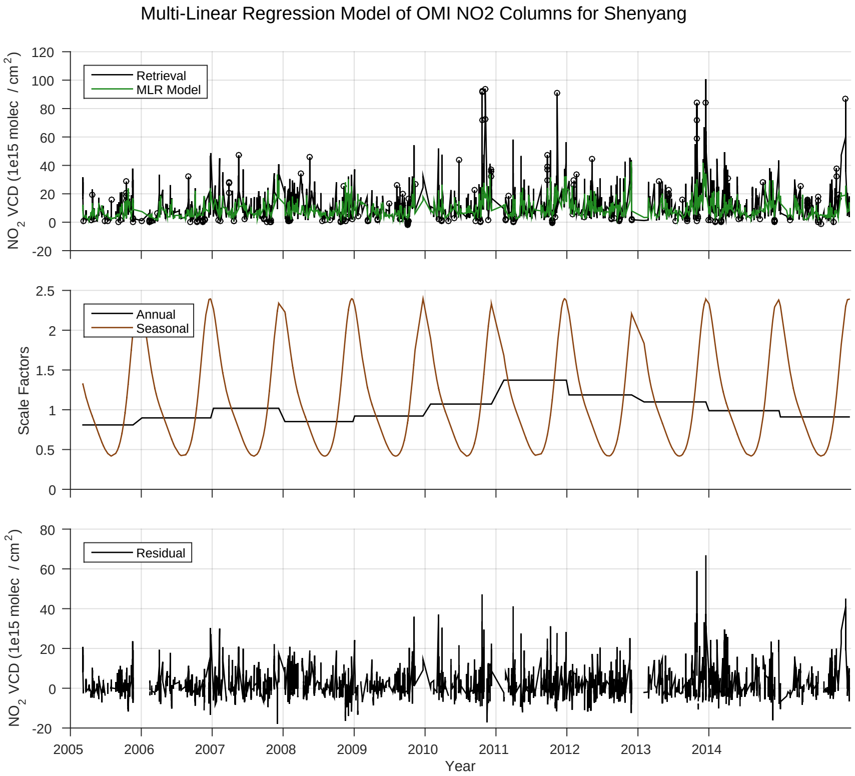

Stats		Annual	%	Weekday	%	Met/Pixel		Seasonal	
Nobs	4735	2005	-19.1	Mon	6.9	WS10	-0.17	Sin 1yr	0.13
Noutliers	229	2006	-10.2	Tue	0.5	T2	0.24	Cos 1yr	0.79
r	0.73	2007	1.9	Wed	0.0	U10	-0.25	Sin 6mo	-0.18
Mean VCD	11.3	2008	-14.9	Thu	3.5	V10	0.43	Cos 6mo	0.06
		2009	-7.9	Fri	-3.9	Dmin	-0.06		
		2010	7.2	Sat	6.6	Dmax	-0.09		
		2011	37.2	Sun	-1.7				
		2012	18.6						
		2013	9.8						
		2014	-1.2						
		2015	-9.0						

Figure S9: Multiple linear regression analysis for Shenyang showing the time series of NO_2 OMI retrievals and the multiple linear regression model fit (top), the annual and seasonal scale factors (middle) and the residual (bottom). Outliers are shown by open circles. The table below the graphs shows the statistics for the model; the percentage difference in NO_2 Vertical Column Density (VCD) for the annual and weekday factors; and multiple linear regression factors for meteorology, pixel resolution and seasonal factors.

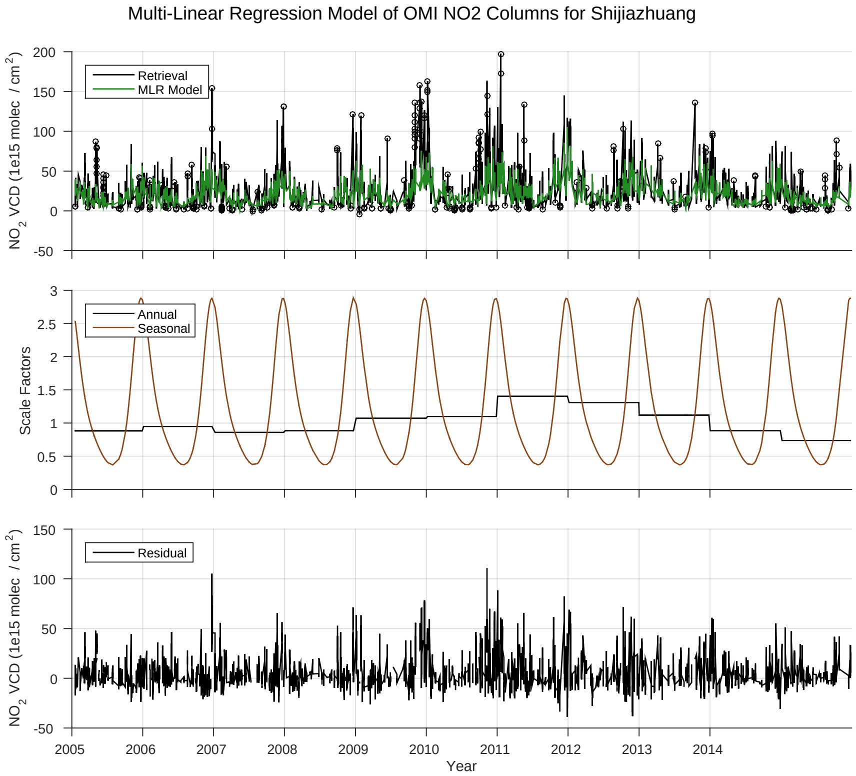

Stats	
Nobs	6365
Noutliers	304
r	0.76
Mean VCD	25.8

Annual	%
2005	-11.9
2006	-5.2
2007	-14.1
2008	-11.6
2009	7.3
2010	9.8
2011	40.4
2012	30.7
2013	12.0
2014	-11.6
2015	-26.3

Weekday	%
Mon	-6.1
Tue	2.2
Wed	0.0
Thu	-2.2
Fri	0.1
Sat	0.2
Sun	-0.1

Met/Pixel	
WS10	-0.18
T2	0.29
U10	-0.02
V10	0.26
Dmin	-0.05
Dmax	-0.14

Seasonal	
Sin 1yr	0.11
Cos 1yr	0.97
Sin 6mo	-0.16
Cos 6mo	0.08

Figure S10: Multiple linear regression analysis for Shijiazhuang showing the time series of NO_2 OMI retrievals and the multiple linear regression model fit (top), the annual and seasonal scale factors (middle) and the residual (bottom). Outliers are shown by open circles. The table below the graphs shows the statistics for the model; the percentage difference in NO_2 Vertical Column Density (VCD) for the annual and weekday factors; and multiple linear regression factors for meteorology, pixel resolution and seasonal factors.

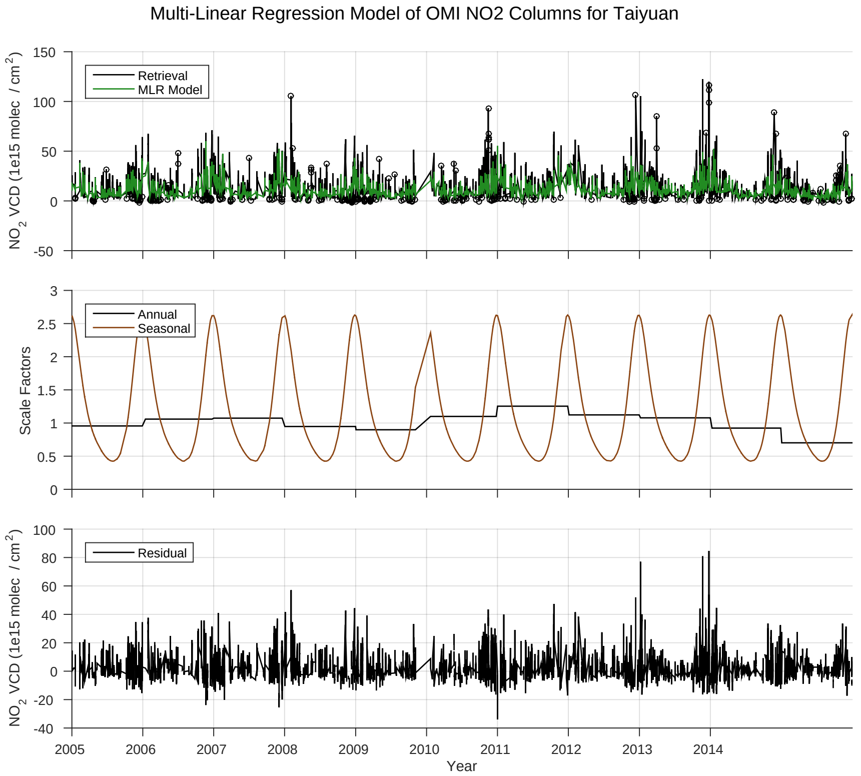

Stats		Annual	%	Weekday	%	Met/Pixel		Seasonal	
Nobs	6522	2005	-4.4	Mon	-4.0	WS10	-0.24	Sin 1yr	0.12
Noutliers	324	2006	5.9	Tue	-0.1	T2	0.21	Cos 1yr	0.86
r	0.67	2007	7.3	Wed	0.0	U10	-0.01	Sin 6mo	-0.11
Mean VCD	13.9	2008	-5.2	Thu	-2.4	V10	0.16	Cos 6mo	0.10
		2009	-10.2	Fri	2.6	Dmin	-0.15		
		2010	10.0	Sat	2.3	Dmax	-0.08		
		2011	25.4	Sun	-7.9				
		2012	12.3						
		2013	7.8						
		2014	-7.7						
		2015	-29.9						

Figure S11: Multiple linear regression analysis for Taiyuan showing the time series of NO_2 OMI retrievals and the multiple linear regression model fit (top), the annual and seasonal scale factors (middle) and the residual (bottom). Outliers are shown by open circles. The table below the graphs shows the statistics for the model; the percentage difference in NO_2 Vertical Column Density (VCD) for the annual and weekday factors; and multiple linear regression factors for meteorology, pixel resolution and seasonal factors.

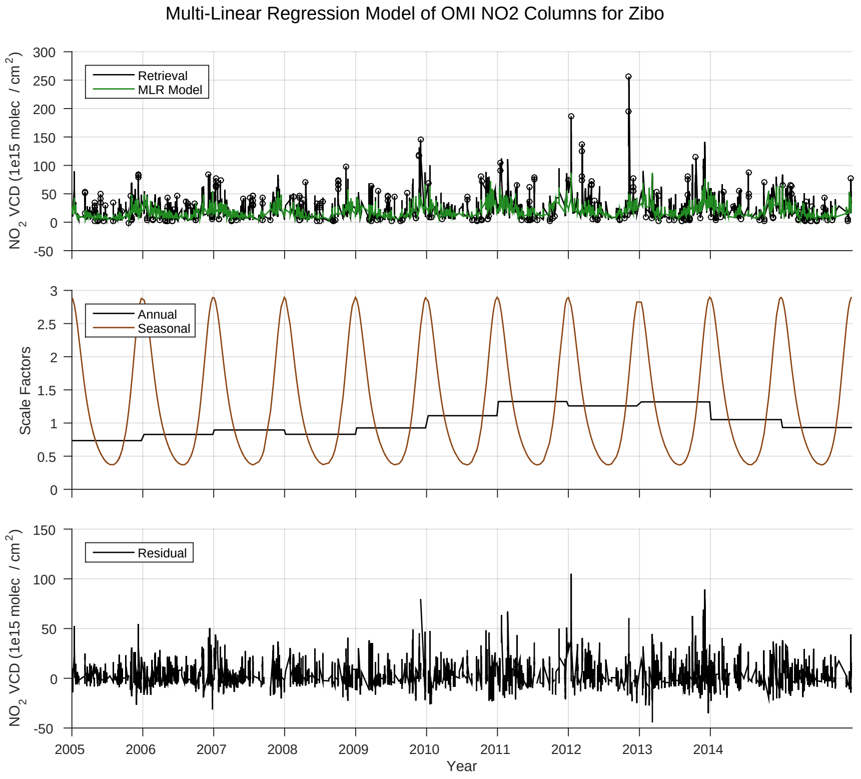

Stats		Annual	%	Weekday	%	Met/Pixel		Seasonal	
Nobs	6449	2005	-26.6	Mon	-3.7	WS10	-0.23	Sin 1yr	0.15
Noutliers	283	2006	-17.2	Tue	-0.5	T2	0.35	Cos 1yr	0.99
r	0.74	2007	-10.5	Wed	0.0	U10	0.05	Sin 6mo	-0.10
Mean VCD	22.5	2008	-17.0	Thu	5.4	V10	0.11	Cos 6mo	0.07
		2009	-7.4	Fri	-4.7	Dmin	-0.17		
		2010	11.0	Sat	0.4	Dmax	-0.02		
		2011	32.4	Sun	-7.9				
		2012	25.8						
		2013	31.9						
		2014	5.1						
		2015	-6.8						

Figure S12: Multiple linear regression analysis for Zibo showing the time series of NO_2 OMI retrievals and the multiple linear regression model fit (top), the annual and seasonal scale factors (middle) and the residual (bottom). Outliers are shown by open circles. The table below the graphs shows the statistics for the model; the percentage difference in NO_2 Vertical Column Density (VCD) for the annual and weekday factors; and multiple linear regression factors for meteorology, pixel resolution and seasonal factors.

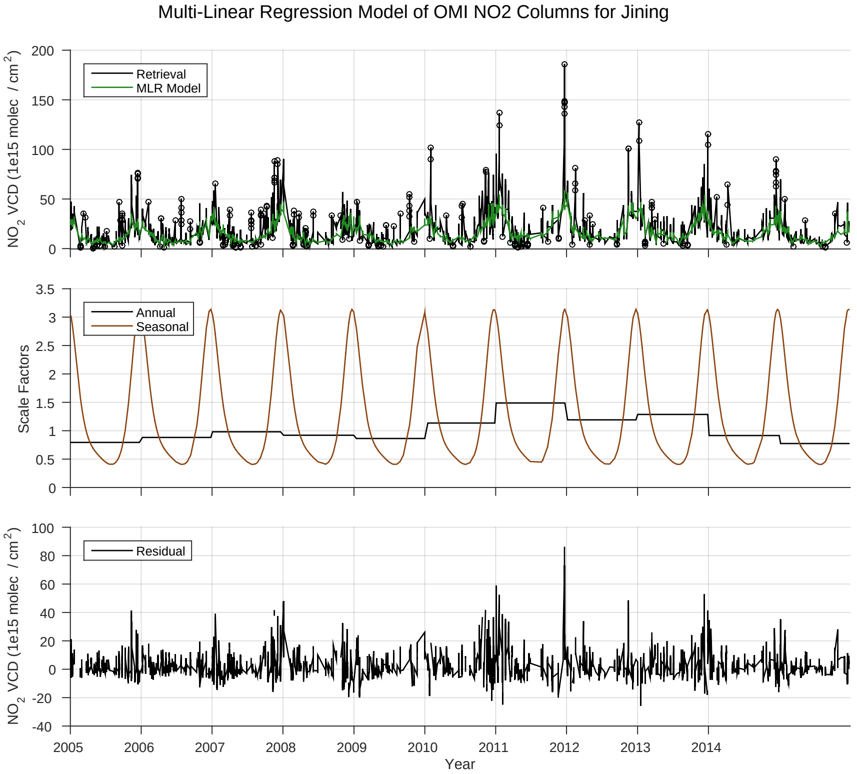

Stats		Annual	%	Weekday	%	Met/Pixel		Seasonal	
Nobs	5128	2005	-20.6	Mon	-4.9	WS10	-0.15	Sin 1yr	0.03
Noutliers	242	2006	-11.9	Tue	-4.7	T2	0.22	Cos 1yr	0.97
r	0.80	2007	-1.9	Wed	0.0	U10	-0.15	Sin 6mo	-0.15
Mean VCD	18.9	2008	-8.0	Thu	2.3	V10	-0.02	Cos 6mo	0.15
		2009	-13.7	Fri	2.6	Dmin	-0.04		
		2010	13.5	Sat	-5.8	Dmax	-0.02		
		2011	48.8	Sun	-6.7				
		2012	19.1						
		2013	28.7						
		2014	-8.5						
		2015	-22.6						

Figure S13: Multiple linear regression analysis for Jining showing the time series of NO_2 OMI retrievals and the multiple linear regression model fit (top), the annual and seasonal scale factors (middle) and the residual (bottom). Outliers are shown by open circles. The table below the graphs shows the statistics for the model; the percentage difference in NO_2 Vertical Column Density (VCD) for the annual and weekday factors; and multiple linear regression factors for meteorology, pixel resolution and seasonal factors.

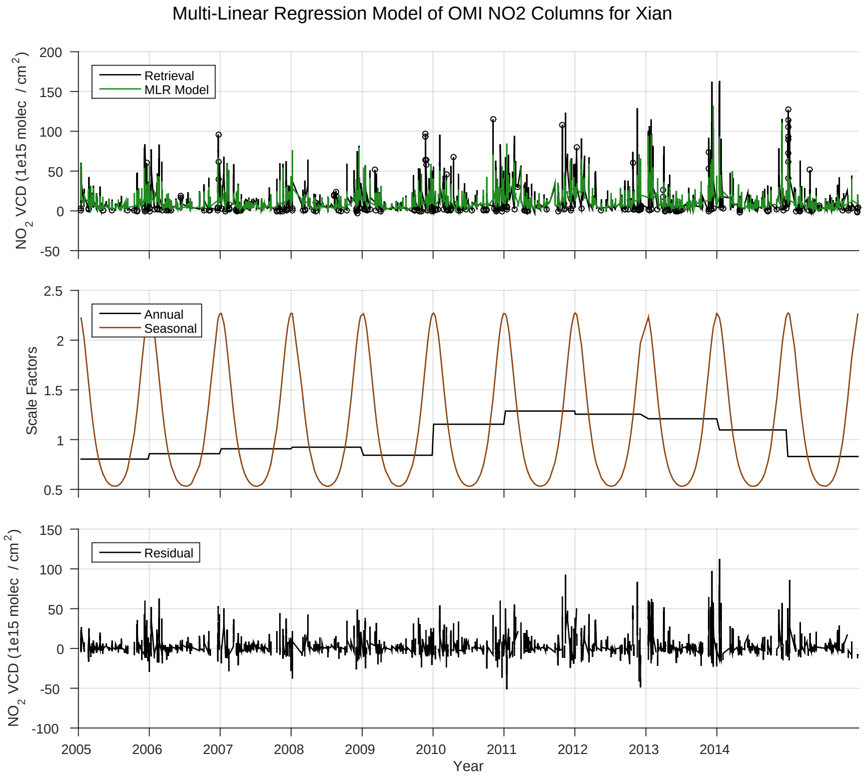

Stats		Annual	%	Weekday	%	Met/Pixel		Seasonal	
Nobs	4690	2005	-19.6	Mon	-4.3	WS10	-0.17	Sin 1yr	0.02
Noutliers	263	2006	-14.2	Tue	0.7	T2	-0.09	Cos 1yr	0.73
r	0.77	2007	-9.2	Wed	0.0	U10	-0.15	Sin 6mo	0.00
Mean VCD	14.9	2008	-7.7	Thu	-7.2	V10	0.25	Cos 6mo	0.09
		2009	-15.7	Fri	7.1	Dmin	-0.25		
		2010	15.4	Sat	-1.3	Dmax	-0.15		
		2011	28.7	Sun	-9.1				
		2012	25.5						
		2013	20.9						
		2014	9.7						
		2015	-17.0						

Figure S14: Multiple linear regression analysis for Xian showing the time series of NO_2 OMI retrievals and the multiple linear regression model fit (top), the annual and seasonal scale factors (middle) and the residual (bottom). Outliers are shown by open circles. The table below the graphs shows the statistics for the model; the percentage difference in NO_2 Vertical Column Density (VCD) for the annual and weekday factors; and multiple linear regression factors for meteorology, pixel resolution and seasonal factors.

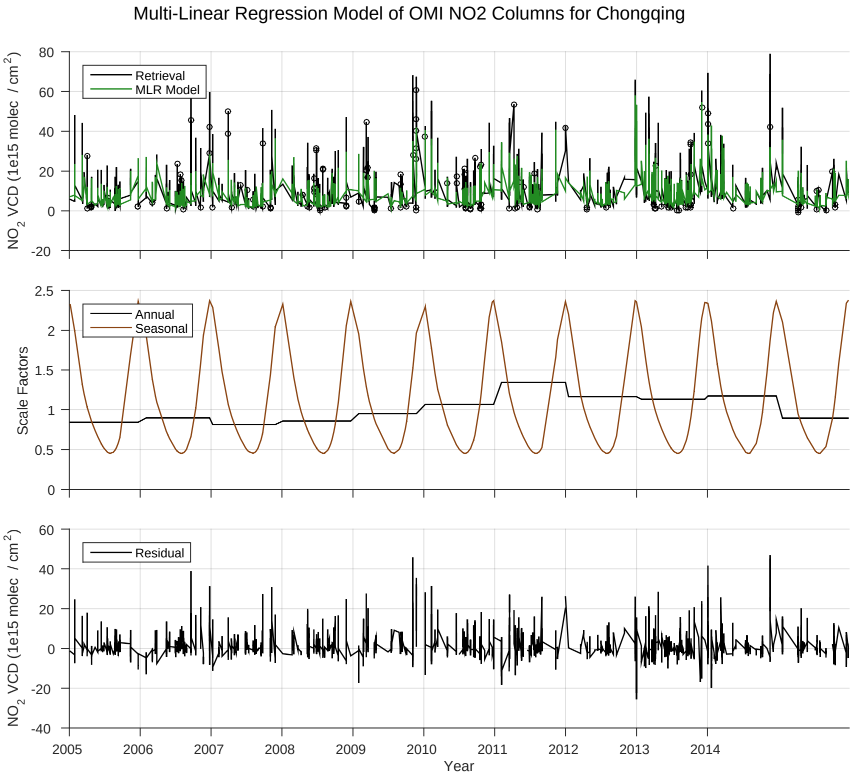

Stats		Annual	%	Weekday	%	Met/Pixel		Seasonal	
Nobs	2941	2005	-15.7	Mon	19.8	WS10	-0.13	Sin 1yr	0.11
Noutliers	152	2006	-10.3	Tue	4.0	T2	0.17	Cos 1yr	0.77
r	0.79	2007	-18.6	Wed	0.0	U10	-0.04	Sin 6mo	-0.13
Mean VCD	10.0	2008	-14.2	Thu	-0.7	V10	-0.04	Cos 6mo	0.08
		2009	-4.9	Fri	-3.1	Dmin	-0.14		
		2010	6.8	Sat	-7.0	Dmax	-0.29		
		2011	34.5	Sun	-4.0				
		2012	16.4						
		2013	13.3						
		2014	17.3						
		2015	-10.5						

Figure S15: Multiple linear regression analysis for Chongqing showing the time series of NO_2 OMI retrievals and the multiple linear regression model fit (top), the annual and seasonal scale factors (middle) and the residual (bottom). Outliers are shown by open circles. The table below the graphs shows the statistics for the model; the percentage difference in NO_2 Vertical Column Density (VCD) for the annual and weekday factors; and multiple linear regression factors for meteorology, pixel resolution and seasonal factors.

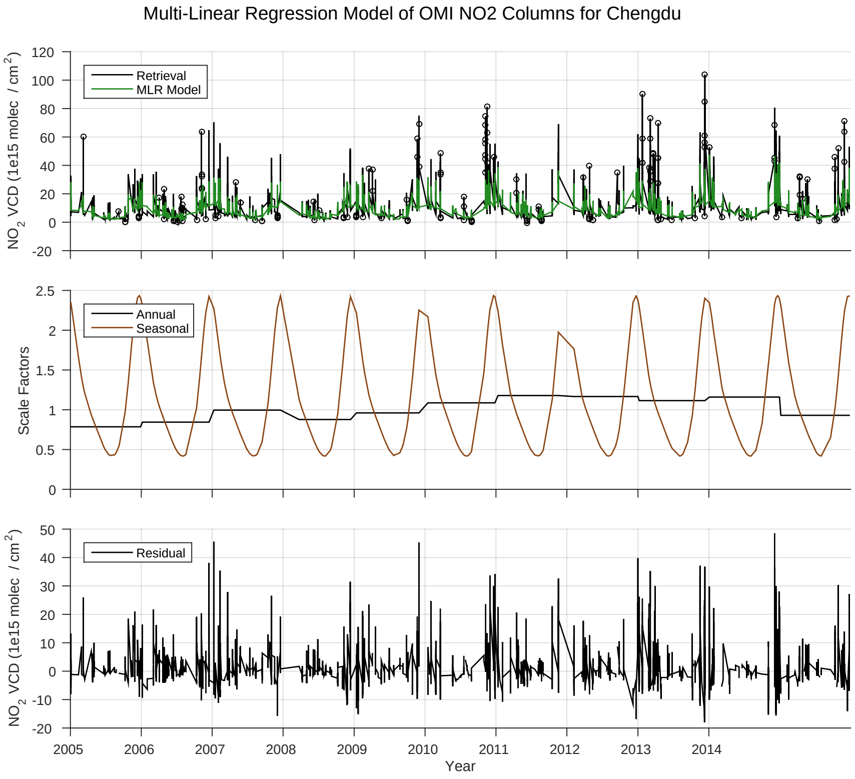

Stats		Annual	%	Weekday	%	Met/Pixel		Seasonal	
Nobs	2995	2005	-21.4	Mon	-15.8	WS10	-0.02	Sin 1yr	0.11
Noutliers	154	2006	-15.6	Tue	-3.3	T2	0.12	Cos 1yr	0.80
r	0.81	2007	-0.4	Wed	0.0	U10	-0.05	Sin 6mo	-0.18
Mean VCD	12.2	2008	-12.3	Thu	3.0	V10	0.01	Cos 6mo	0.07
		2009	-3.9	Fri	0.4	Dmin	-0.12		
		2010	8.7	Sat	-2.7	Dmax	-0.18		
		2011	17.9	Sun	-16.9				
		2012	16.6						
		2013	11.5						
		2014	16.0						
		2015	-7.0						

Figure S16: Multiple linear regression analysis for Chengdu showing the time series of NO_2 OMI retrievals and the multiple linear regression model fit (top), the annual and seasonal scale factors (middle) and the residual (bottom). Outliers are shown by open circles. The table below the graphs shows the statistics for the model; the percentage difference in NO_2 Vertical Column Density (VCD) for the annual and weekday factors; and multiple linear regression factors for meteorology, pixel resolution and seasonal factors.

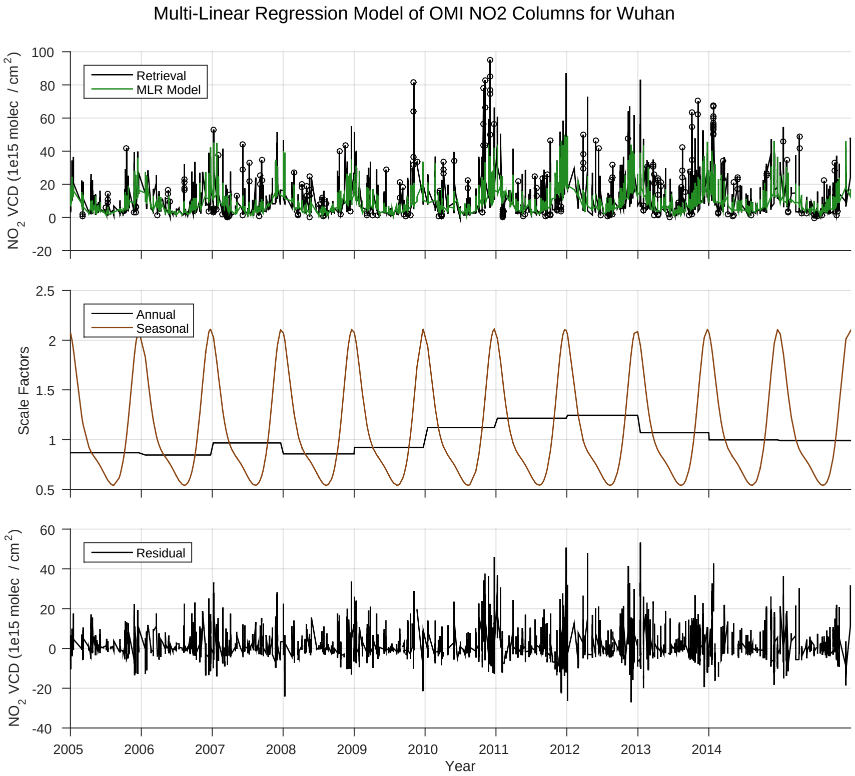

Stats		Annual	%	Weekday	%	Met/Pixel		Seasonal	
Nobs	4905	2005	-13.2	Mon	-2.6	WS10	-0.17	Sin 1yr	0.07
Noutliers	251	2006	-15.5	Tue	2.4	T2	-0.05	Cos 1yr	0.60
r	0.81	2007	-3.4	Wed	0.0	U10	-0.14	Sin 6mo	-0.13
Mean VCD	11.9	2008	-14.4	Thu	1.3	V10	-0.06	Cos 6mo	0.13
		2009	-7.9	Fri	-3.6	Dmin	-0.22		
		2010	12.1	Sat	-4.4	Dmax	-0.06		
		2011	21.5	Sun	-3.2				
		2012	24.4						
		2013	7.0						
		2014	-0.3						
		2015	-1.1						

Figure S17: Multiple linear regression analysis for Wuhan showing the time series of NO_2 OMI retrievals and the multiple linear regression model fit (top), the annual and seasonal scale factors (middle) and the residual (bottom). Outliers are shown by open circles. The table below the graphs shows the statistics for the model; the percentage difference in NO_2 Vertical Column Density (VCD) for the annual and weekday factors; and multiple linear regression factors for meteorology, pixel resolution and seasonal factors.

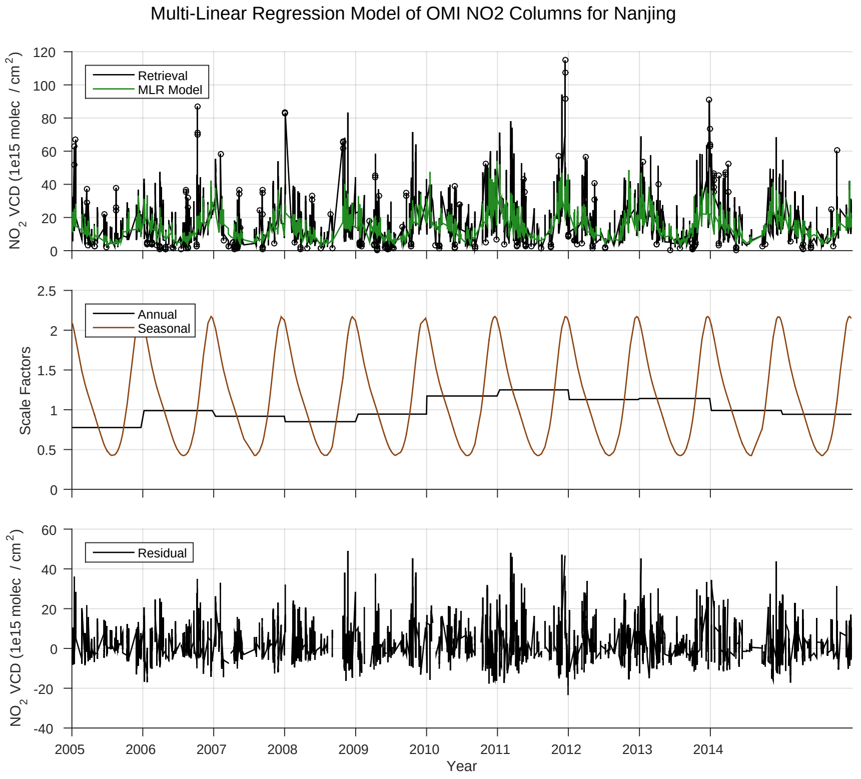

Stats		Annual	%	Weekday	%	Met/Pixel		Seasonal	
Nobs	5024	2005	-22.3	Mon	-2.1	WS10	-0.13	Sin 1yr	0.12
Noutliers	234	2006	-1.1	Tue	-7.1	T2	0.09	Cos 1yr	0.74
r	0.73	2007	-8.2	Wed	0.0	U10	-0.13	Sin 6mo	-0.18
Mean VCD	17.4	2008	-15.0	Thu	6.4	V10	0.12	Cos 6mo	0.00
		2009	-5.5	Fri	1.2	Dmin	-0.09		
		2010	17.3	Sat	-5.4	Dmax	-0.07		
		2011	25.0	Sun	-6.1				
		2012	12.8						
		2013	14.2						
		2014	-0.9						
		2015	-5.7						

Figure S18: Multiple linear regression analysis for Nanjing showing the time series of NO_2 OMI retrievals and the multiple linear regression model fit (top), the annual and seasonal scale factors (middle) and the residual (bottom). Outliers are shown by open circles. The table below the graphs shows the statistics for the model; the percentage difference in NO_2 Vertical Column Density (VCD) for the annual and weekday factors; and multiple linear regression factors for meteorology, pixel resolution and seasonal factors.

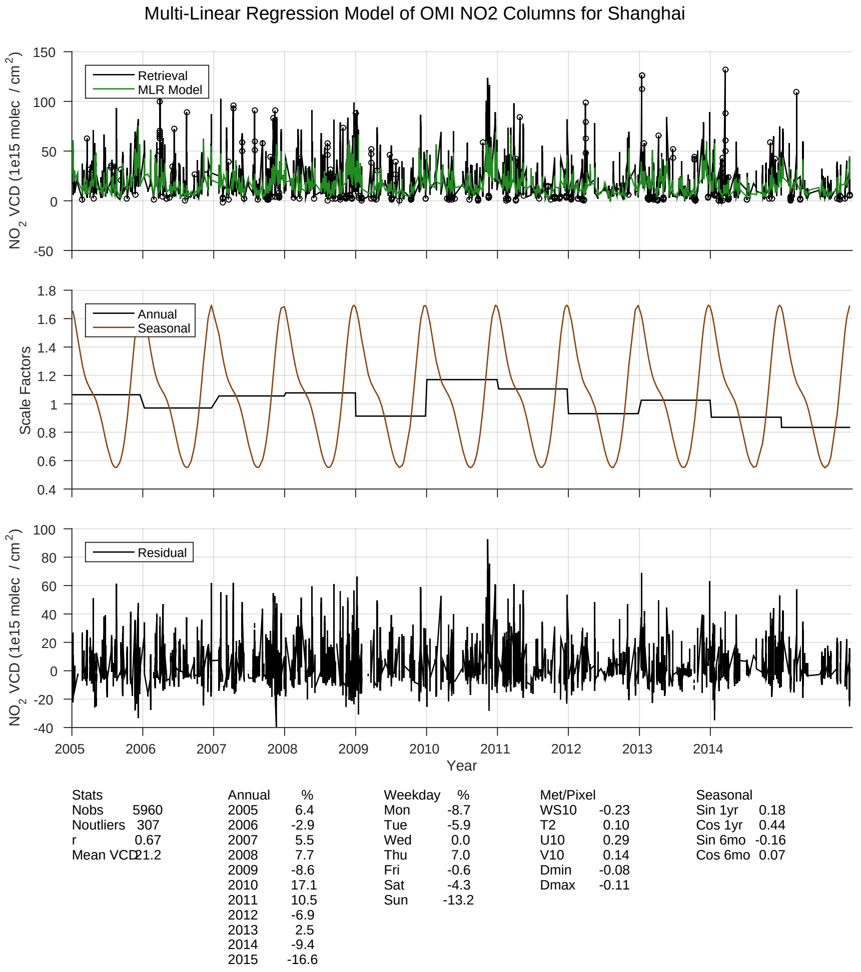

Figure S19: Multiple linear regression analysis for Shanghai showing the time series of NO_2 OMI retrievals and the multiple linear regression model fit (top), the annual and seasonal scale factors (middle) and the residual (bottom). Outliers are shown by open circles. The table below the graphs shows the statistics for the model; the percentage difference in NO_2 Vertical Column Density (VCD) for the annual and weekday factors; and multiple linear regression factors for meteorology, pixel resolution and seasonal factors.

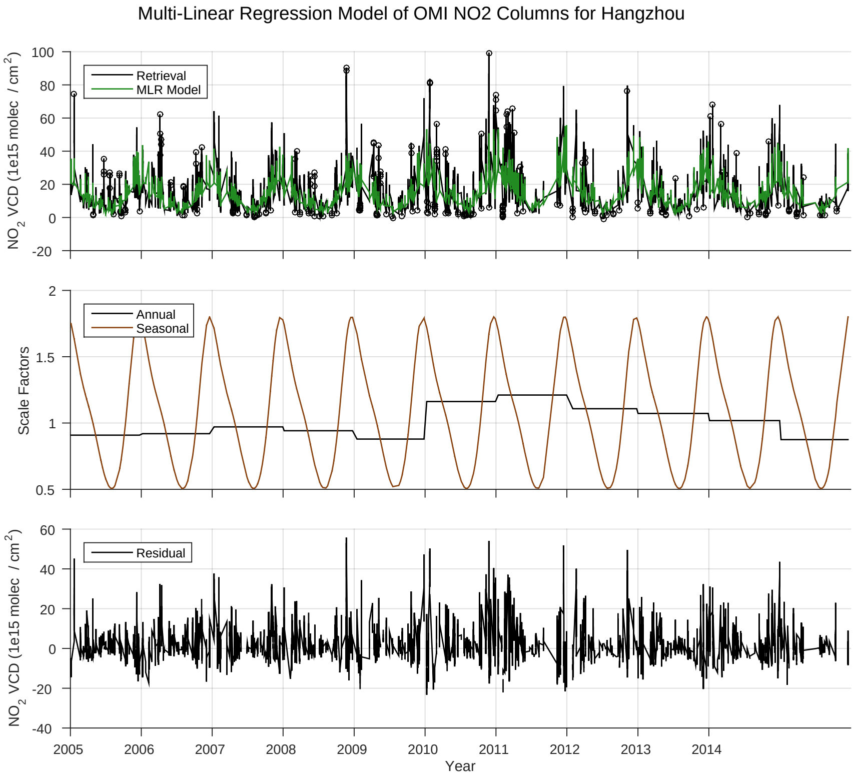

Stats		Annual	%	Weekday	%	Met/Pixel		Seasonal	
Nobs	5306	2005	-9.2	Mon	-4.2	WS10	-0.15	Sin 1yr	0.14
Noutliers	255	2006	-8.0	Tue	-2.7	T2	-0.04	Cos 1yr	0.56
r	0.80	2007	-2.9	Wed	0.0	U10	-0.08	Sin 6mo	-0.15
Mean VCD	17.2	2008	-5.8	Thu	3.2	V10	-0.17	Cos 6mo	0.01
		2009	-12.1	Fri	-0.4	Dmin	-0.07		
		2010	16.2	Sat	-0.5	Dmax	-0.11		
		2011	21.1	Sun	-0.0				
		2012	10.8						
		2013	7.2						
		2014	1.8						
		2015	-12.5						

Figure S20: Multiple linear regression analysis for Hangzhou showing the time series of NO_2 OMI retrievals and the multiple linear regression model fit (top), the annual and seasonal scale factors (middle) and the residual (bottom). Outliers are shown by open circles. The table below the graphs shows the statistics for the model; the percentage difference in NO_2 Vertical Column Density (VCD) for the annual and weekday factors; and multiple linear regression factors for meteorology, pixel resolution and seasonal factors.

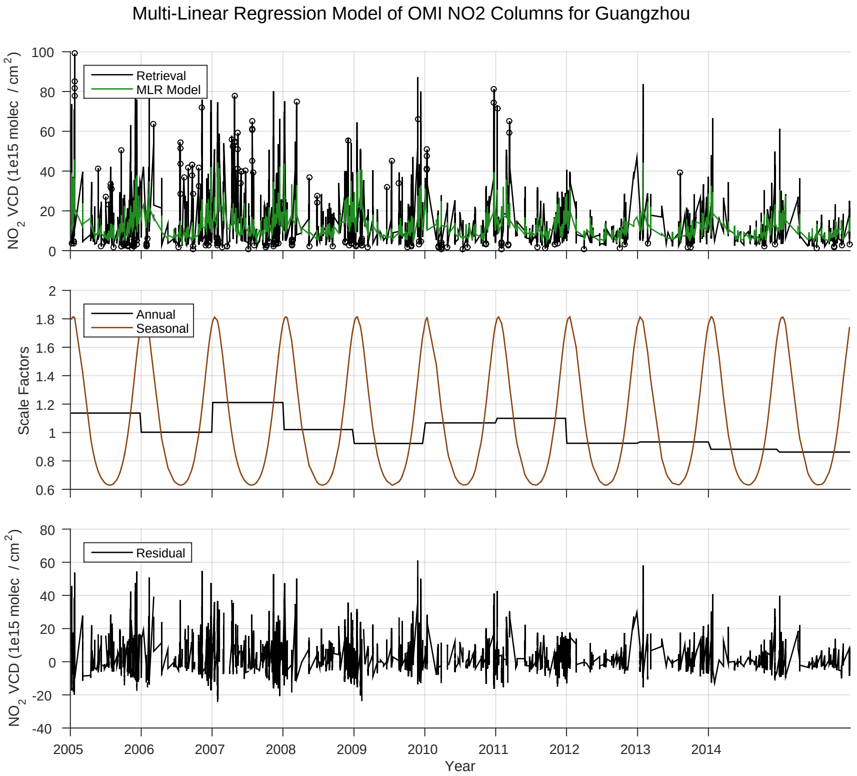

Stats	
Nobs	4530
Noutliers	197
r	0.61
Mean VCD	15.8

Annual	%
2005	13.7
2006	0.2
2007	21.1
2008	2.0
2009	-7.7
2010	6.8
2011	10.0
2012	-7.6
2013	-6.7
2014	-11.8
2015	-13.8

Weekday	%
Mon	-13.6
Tue	-0.5
Wed	0.0
Thu	-5.1
Fri	6.0
Sat	-7.4
Sun	-12.3

Met/Pixel	
WS10	-0.18
T2	0.04
U10	0.01
V10	0.08
Dmin	-0.06
Dmax	-0.12

Seasonal	
Sin 1yr	0.14
Cos 1yr	0.51
Sin 6mo	0.03
Cos 6mo	0.06

Figure S21: Multiple linear regression analysis for Guangzhou showing the time series of NO_2 OMI retrievals and the multiple linear regression model fit (top), the annual and seasonal scale factors (middle) and the residual (bottom). Outliers are shown by open circles. The table below the graphs shows the statistics for the model; the percentage difference in NO_2 Vertical Column Density (VCD) for the annual and weekday factors; and multiple linear regression factors for meteorology, pixel resolution and seasonal factors.

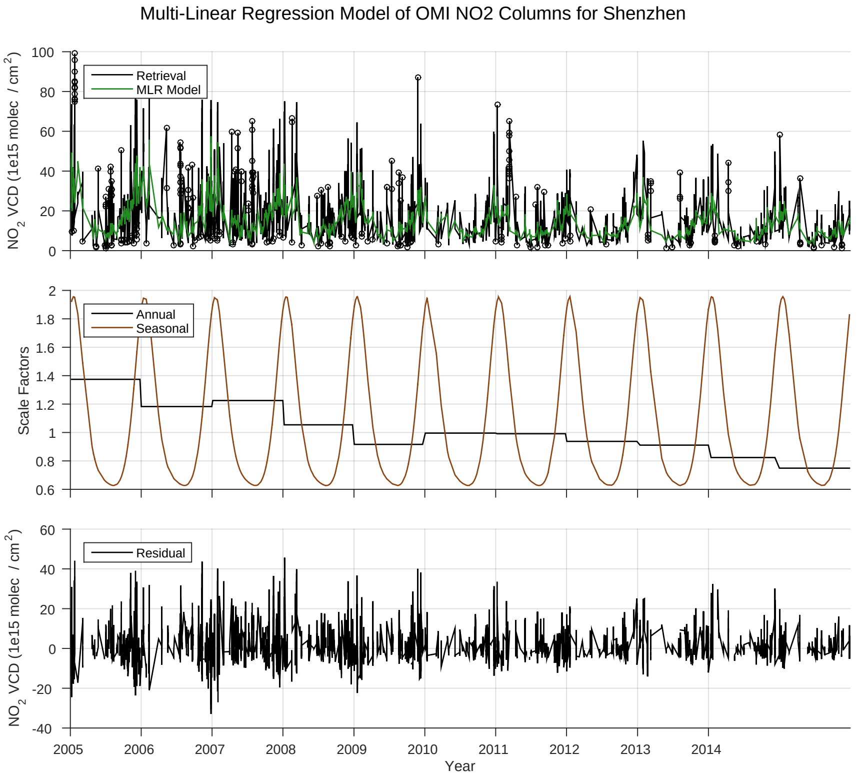

Stats	
Nobs	4691
Noutliers	246
r	0.78
Mean VCD	18.5

Annual	%
2005	37.4
2006	18.3
2007	22.5
2008	5.4
2009	-8.4
2010	-0.4
2011	-0.9
2012	-6.3
2013	-8.9
2014	-17.6
2015	-25.1

Weekday	%
Mon	-12.6
Tue	4.5
Wed	0.0
Thu	-5.2
Fri	1.0
Sat	-3.1
Sun	-19.0

Met/Pixel	
WS10	-0.29
T2	0.10
U10	-0.08
V10	-0.12
Dmin	-0.07
Dmax	0.01

Seasonal	
Sin 1yr	0.20
Cos 1yr	0.53
Sin 6mo	0.05
Cos 6mo	0.10

Figure S22: Multiple linear regression analysis for Shenzhen showing the time series of NO_2 OMI retrievals and the multiple linear regression model fit (top), the annual and seasonal scale factors (middle) and the residual (bottom). Outliers are shown by open circles. The table below the graphs shows the statistics for the model; the percentage difference in NO_2 Vertical Column Density (VCD) for the annual and weekday factors; and multiple linear regression factors for meteorology, pixel resolution and seasonal factors.

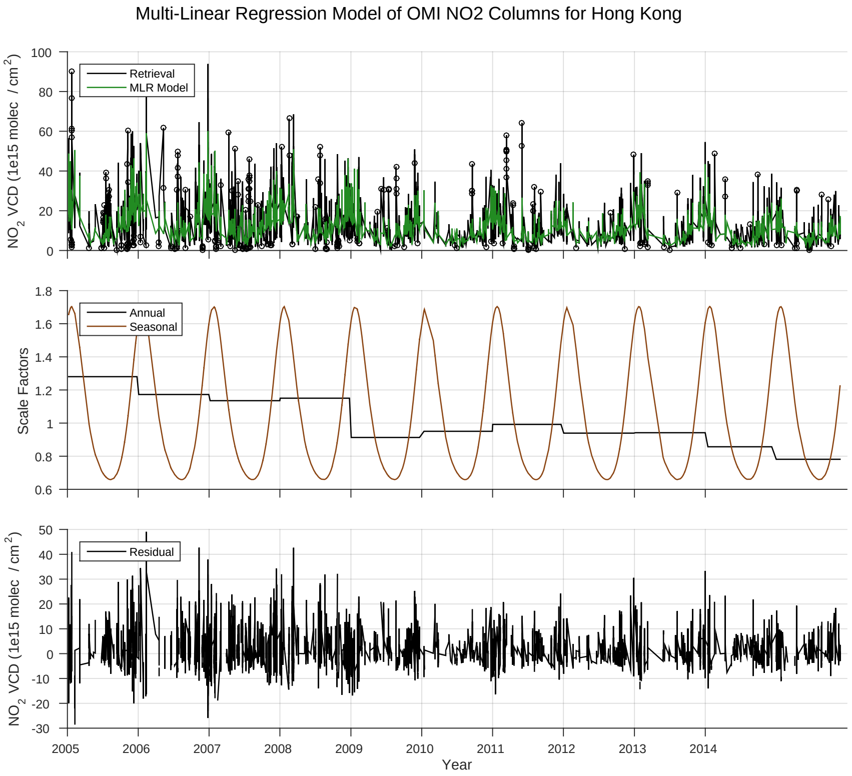

Stats		Annual	%	Weekday	%	Met/Pixel		Seasonal	
Nobs	4847	2005	28.0	Mon	-14.6	WS10	-0.25	Sin 1yr	0.21
Noutliers	264	2006	17.3	Tue	4.0	T2	0.09	Cos 1yr	0.42
r	0.75	2007	13.5	Wed	0.0	U10	0.05	Sin 6mo	0.03
Mean VCD	15.0	2008	15.0	Thu	-3.3	V10	-0.31	Cos 6mo	0.06
		2009	-8.7	Fri	-0.6	Dmin	-0.08		
		2010	-5.0	Sat	-13.0	Dmax	-0.15		
		2011	-0.8	Sun	-22.7				
		2012	-6.1						
		2013	-5.8						
		2014	-14.3						
		2015	-21.9						

Figure S23: Multiple linear regression analysis for showing the time series of NO_2 OMI retrievals and the multiple linear regression model fit (top), the annual and seasonal scale factors (middle) and the residual (bottom). Outliers are shown by open circles. The table below the graphs shows the statistics for the model; the percentage difference in NO_2 Vertical Column Density (VCD) for the annual and weekday factors; and multiple linear regression factors for meteorology, pixel resolution and seasonal factors.
